# Oxidative stress-triggered UMPylation of SodA by YdiU modulates oxidative stress resistance in *Salmonella*

**DOI:** 10.1186/s13567-026-01818-7

**Published:** 2026-07-11

**Authors:** Nannan Song, Li Zhai, Yingying Yue, Weiwei Wang, Cuiling Li, Qing Liu, Xinyi Wang, Yanan Wang, Zihan Shao, Junyong Yang, Zhaotai Zang, Bingqing Li, Haihong Jia

**Affiliations:** 1 Department of Clinical Laboratory, Shandong Provincial Hospital Affiliated to Shandong First Medical University, Jinan, 250000 China; 2 Department of Pathogen Biology, School of Basic Medicine, Shandong First Medical University and Shandong Academy of Medical Sciences, Jinan, 250000 China; 3 Key Lab for Biotech-Drugs of National Health Commission, Jinan, 250000 China; 4 Key Lab for Rare and Uncommon Diseases of Shandong Province, Jinan, 250000 China

**Keywords:** *Salmonella*, YdiU, SodA, UMPylation, oxidation balance

## Abstract

YdiU is a novel UMPylation modification enzyme that plays an important role in regulating chaperones, flagellar synthesis, and iron absorption in *Salmonella*. Here, we report that YdiU plays an important role in regulating oxidative stress in *Salmonella*. The expression of *ydiU* was dramatically induced by hydrogen peroxide (H_2_O_2_) and the survivability of ∆*ydiU* was significantly stronger than that of WT under oxidative stress. Interestingly, in vitro and in vivo experiments showed that YdiU can interact with SodA and UMPylate it only in the presence of H_2_O_2_. Further study showed slight changes in the secondary structure of SodA under oxidative stress. Structural analysis of native protein and H_2_O_2_-treated protein showed that H_2_O_2_ treatment affected the conformation of Met24. The modification site Tyr12 is near Met24, and Tyr12 exposure may facilitate UMPylation by YdiU after H_2_O_2_ treatment. In addition, in vivo and in vitro experiments showed that YdiU negatively regulates SodA activity. Therefore, our results identify new biological functions of YdiU, highlight the importance of UMPylation in bacterial oxidation balance, and provide the theoretical basis to study *Salmonella* survival and pathogenesis.

**Supplementary Information:**

The online version contains supplementary material available at 10.1186/s13567-026-01818-7.

## Introduction

*Salmonella* Typhimurium is an important pathogenic bacterium that can cause infections in various animals, especially domestic livestock such as pigs, cattle, sheep, and poultry. After infection, it may lead to symptoms such as diarrhea, fever, and dehydration, and in severe cases, it can cause death, resulting in economic losses to the animal husbandry industry [1]. *Salmonella* can exhibit drug resistance, limiting conventional drug [2–4]. Therefore, an increased interest to understand *Salmonella* pathology in the fields of medicine, veterinary medicine and public health. As facultative intracellular bacteria, *Salmonella* can survive, replicate, and disseminate in host macrophages to evade host immune clearance [4, 5]. The formation of *Salmonella*-containing vesicles (SCV) in the cytoplasm provides a safe and stable parasitic environment for the *Salmonella*, ensuring their persistence in the host [6]. However, *Salmonella* faces a variety of survival pressures in SCV, such as pro-inflammatory cytokines, antimicrobial peptides, and reactive oxygen species (ROS) [7–9]. In order to adapt to its environment, *Salmonella* has evolved various mechanisms to evade the host immune system and defend against harmful substances such as oxygen free radicals produced by the host. However, the survival mechanisms of *Salmonella* are complex and poorly understood.

After invading, *Salmonella* encounters a high ROS internal environment formed by the host for immune protection [7]. To survive oxidative conditions and establish the infectious process, *Salmonella* has evolved a variety of strategies to protect the bacteria from ROS damage, including (1) Interference with NADPH oxidase assembly to reduce ROS production [10]; (2) Produce protein and DNA repair enzymes to repair damaged molecules [11, 12]; (3) Action of multiple antioxidant enzymes to protect bacteria from oxidative stress [10]. The antioxidant enzyme system of *Salmonella* is very complex, and includes catalase (CAT), superoxide dismutase (SOD), and methionine sulfoxide reductase A (MsrA) [13]. SOD scavenges superoxide anions (O_2_⁻) and converts them into H_2_O_2_, while CAT degrades H_2_O_2_, forming an upstream stepwise ROS scavenging defense. MsrA, a protein repair enzyme, maintains SOD and CAT activity by repairing ROS-induced oxidized methionine residues and protects key intracellular proteins. The three synergize to form a “ROS detoxification-repair” regulatory network, mediating STM’s resistance to oxidative stress [11, 14, 15]. Among them, SODs are the only enzymes that specifically interact with superoxide to control the levels of ROS and serve as key regulators of signaling [16]. There are three main types of SOD_S_ in bacterial pathogens: SodA, SodB and SodC, which rely on Mn, Fe, and Cu–Zn ions, respectively [17]. Most studies of the regulation of SOD activity have focused on changes at transcriptional and translational levels. However, few studies have examined the effects of post-translational modification on this process.

YdiU belongs to the ubiquitous UPF0061 family, which is highly conserved across diverse bacteria and thus assumed to perform indispensable physiological functions. As a conserved hypothetical protein with uncharacterized function, this family is recognized as one of the top ten high-priority “unknown unknowns” targets, endowing YdiU with important research significance [18]. However, understanding of YdiU is still in the initial stage, with many knowledge gaps need to be filled. Recent work showed that YdiU belongs to the widespread and highly conserved SelO family and catalyzes AMPylation and UMPylation [18]. Sreelatha et al. reported YdiU as an AMPylator that can transfer AMP from ATP to Ser, Thr, and Tyr residues on protein substrates [19]. It was found in our laboratory that YdiU could not only catalyze AMPylation, but also transfer UMP from UTP to His and Tyr of protein [20]. We identified YdiU as an unusual UMPylation modification enzyme, and we found 46 UMPylated bacterial proteins (including SodA) in a YdiU-expressing *Salmonella* strain by spectrometry-based proteomic analysis [20]. *Salmonella* SodA belongs to the Mn superoxide dismutase family related to oxidative stress [21], but it was unknown if the modification of SodA by YdiU could affect the antioxidant capacity of *Salmonella*.

In this study, we report here that YdiU can regulate oxidative stress in *Salmonella*. YdiU expression was dramatically induced by hydrogen peroxide (H_2_O_2_). Growth curve and survival rate data showed that *Salmonella* YdiU-deficient strain (Δ*ydiU*) exhibited stronger antioxidant capacity under H_2_O_2_ pressure. To further elucidate the mechanism by which YdiU regulates bacterial antioxidant capacity through modification of SodA protein, we detected the interaction between YdiU and H_2_O_2_ treated SodA by bacterial two-hybrid assay and protein cross-linking assay. YdiU efficiently catalyzed the UMPylation of SodA under oxidative stress, and we identified Tyr12 of SodA by mass spectrometry as the site of UMPylation. The protein structures of native SodA and 0.15 mM H_2_O_2_-treated SodA revealed obvious structural phase migration of Met24 of SodA under oxidative stress. Importantly, the enzyme activity of SodA decreased after YdiU modification. Together, our results illuminate new biological functions of YdiU, highlight the importance of UMPylation in bacterial signal transduction, and reveal a potential mechanism by which bacteria adapt to oxidative stress environment.

## Materials and methods

### Bacterial strains and culture conditions

**Figure 1:**
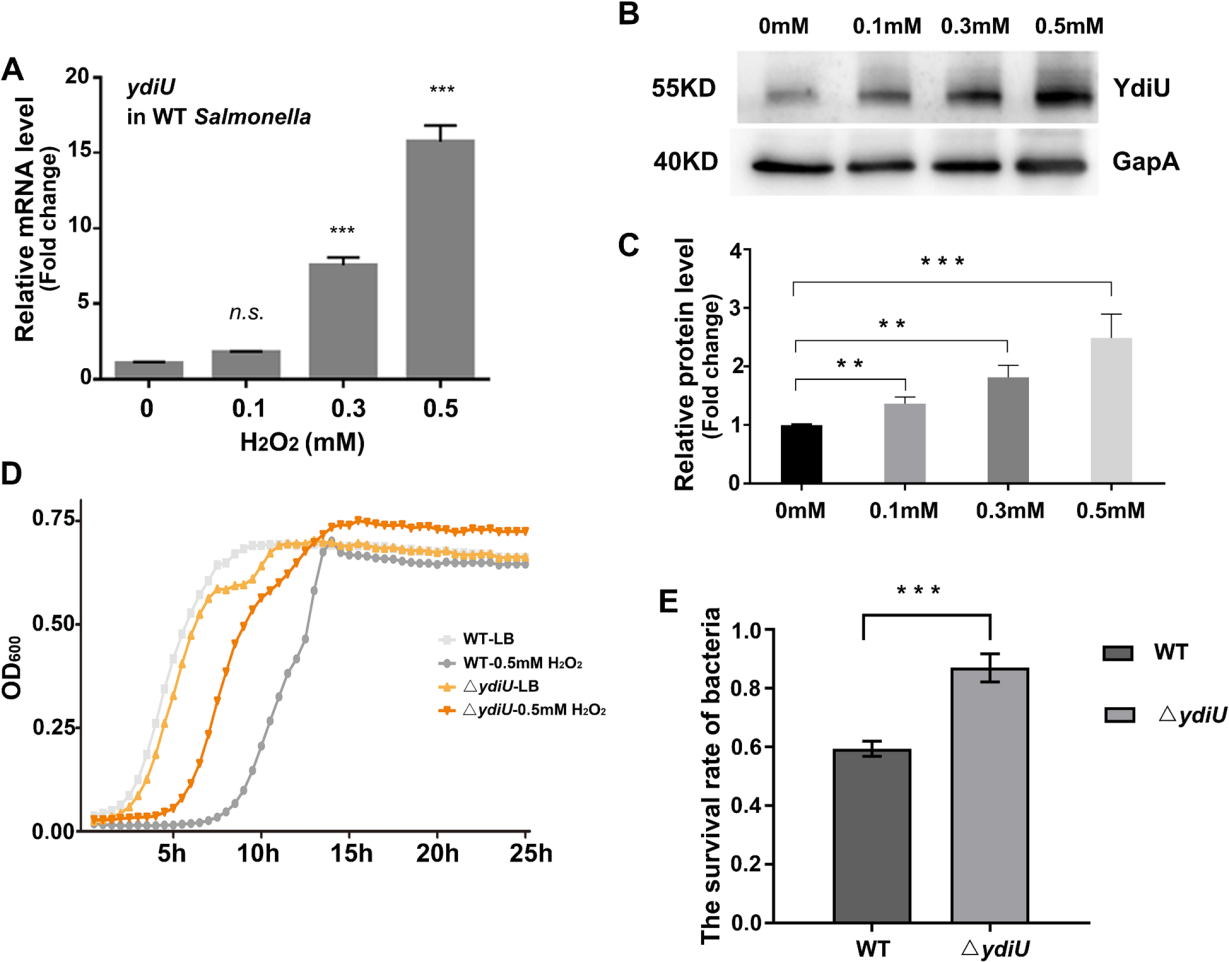
**YdiU is involved in the regulation of oxidative stress**. **A**, **B** The transcription and protein levels of YdiU in *Salmonella* cultivated with different concentrations (0, 0.1, 0.3, 0.5 mM) of H_2_O_2_ were detected with qRT-PCR and western blotting, respectively. GapA (also known as glyceraldehyde-3-phosphate dehydrogenase [GAPDH]) was used as a loading control. **C** Relative expression levels of target protein determined by WB gray intensity analysis. The band gray values of western blot were quantitatively analyzed, and the expression fold change was normalized to the 0 mM control group. **D** The growth curves of WT and Δ*ydiU* cultivated in LB medium or 0.5 mM H_2_O_2_ conditions. **E** The survival rates of WT and Δ*ydiU* strains under 0.5 mM H_2_O_2_ pressure. The above-described experiments were performed as three replicates, and the mean values are presented. One-way ANOVA was used for statistical analysis. ***P < 0.001; **P < 0.01; *P < 0.05.

All bacterial strains of *S.* Typhimurium and *E. coli* used in this study are listed in Additional file 1 in the supplemental material. Generally, the strains were grown in LB medium or cultivated on LB agar plates containing 1.5% (wt/vol) agar supplemented with 100 μg/mL ampicillin or 50 μg/mL kanamycin as required at 37 °C. For oxidant survival experiments, the strains were activated in LB medium overnight, and then transferred to fresh medium for homogenized growth at an optical density at 600 nm (OD_600_) of 0.01. Different concentrations of H_2_O_2_ were added when strains entered mid-log phase (OD_600_ = 0.5). For the experiments shown in Figure 1A, B, and Additional file 5A, B, *Salmonella* cultures entered mid-log phase and were treated with different concentrations of H_2_O_2_ (0, 0.1, 0.3, 0.5 mM) for 2 h to induce gene expression.

### Generation of recombinant plasmids

The primers and plasmids used in this study are listed in Additional file 2 and Additional file 3. The *sodA* gene was amplified from *E. coli* strain K-12 substrain MG1655 genomic DNA and cloned into the pGL01 vector for biochemical study. For the bacterial two-hybrid assay, *sodA* was cloned into pKNT25. Other plasmids (Vector pKNT25 control, Kan^+^; Vector pUT18C control, Amp^+^; zip/pKNT25, Kan^+^; zip/pUT18C, Amp^+^; *ydiU*/pUT18C, Amp^+^) used the BACTH assay were previously constructed and stored in our laboratory [20, 22–25].

### Protein expression and purification

The *sodA* open reading frame (ORF) was amplified using primers *sodA*-pGL01-F and *sodA*-pGL01-R (Additional file 2) and cloned into the pGL01 vector. The recombinant plasmids (SodA/pGL01 and YdiU475/pGL01) were transformed into *Escherichia coli* BL21 (DE3) for protein expression. Cells were cultured in ampicillin-supplemented LB medium at 37 °C to an optical density (OD_600_) of 0.6. For native SodA expression, the culture was cooled to 16 °C for 1 h, followed by induction with 0.4 mM IPTG overnight. For SodA expression under H_2_O_2_ stress, cultures at an OD_600_ of 0.6 were pre-treated with 0.1 mM or 0.5 mM H_2_O_2_ for 3 h prior to the 16 °C cooling and overnight IPTG induction. Cells were harvested by centrifugation (4000 rpm, 18 min, 4 °C) and resuspended in lysis buffer (25 mM Tris–HCl, pH 8.0, 200 mM NaCl) supplemented with PMSF. After sonication on ice, the lysate was centrifuged to collect the soluble supernatant. The supernatant was loaded onto a pre-equilibrated Ni–NTA affinity column. Following a wash step with the lysis buffer, on-column cleavage was performed overnight at 4 °C using PPase, and the target protein was eluted. The eluate was concentrated to 2 mL and further purified by size-exclusion chromatography (AKTA system) using SD buffer (10 mM Tris–HCl, pH 8.0, 100 mM NaCl). Protein purity across all extraction and purification steps was monitored by 12% SDS-PAGE and Coomassie brilliant blue staining. Finally, fractions containing highly pure SodA were pooled, quantified, flash-frozen in liquid nitrogen, and stored at −80 °C.

### Disc diffusion assay

The two plasmids [SodA/pGL01 and pET29b, SodA/pGL01 and YdiU^475^/pET29b (3T)) were co-transferred into BL21 to obtain a co-expressing strain. To analyze the enzyme activity of SodA before and after UMPylation, disc diffusion assays were performed as described [24]. In short, approximately 5 × 10^8^ *E. coli* cells were overlaid onto LB-ampicillin/kanamycin agar and incubated at 37 °C for 1 h. Filter discs (6 mm diameter) soaked in different concentrations of H_2_O_2_ (0, 1, 2, 2.5, and 5 mM) were placed on the agar surface. After incubation at 37 °C for 12 h, the killing zones around the paper discs were photographed and measured as described [25].

### RNA extraction and real-time quantitative PCR

Total RNA was extracted from treated *Salmonella* using the SPARKeasy bacterial/cell RNA kit (Sparkjade, Shandong, China) according to the manufacturer’s instructions. Then, 2 μg of total RNA was used for reverse transcription reactions using HiScript III RT SuperMix (Vazyme, Nanjing, China), with the product used as a qRT-PCR (quantitative real-time PCR) template. GAPDH (glyceraldehyde-3-phosphate dehydrogenase, also termed GapA) was chosen as the internal control for relative transcript quantification instead of 16S rRNA. As a key glycolytic enzyme, GapA displays stable expression in *Salmonella* upon ROS stress, and its single-copy status in the bacterial genome avoids copy-number bias inherent to multicopy 16S rRNA, thus ensuring higher reliability in mRNA quantification. The TB Green Premix Ex Taq II (TaKaRa, Japan) and Applied Biosystems 7500 Sequence Detection system (Applied Biosystems, Foster, CA, USA) were used for qRT-PCR. The PCR mixture and reaction conditions were as previously described [26]. Relative expression data were automatically analyzed using system’s software, and the expression levels were calculated using the 2^−ΔΔCt^ comparative CT method [27]. The primers used for qRT-PCR are listed in Additional file 2.

### Protein extraction and western blot

Total protein was extracted from bacterial cells subjected to different concentrations of H_2_O_2_ and examined by 12% SDS-PAGE. The antibody to GapA was used as the reference. Antibodies against YdiU and SodA were prepared by Dia-An Biotech, Inc. (Wuhan, China). Western blot was performed as previously described [23]. In short, total proteins were separated by 12% SDS-PAGE and then electrotransferred onto a polyvinylidene fluoride (PVDF) membrane (Millipore, Bedford, MA). The membrane was blotted with polyclonal antibodies developed against GapA, YdiU, SodA and biotin-labelled goat anti-Rabbit IgG (Abcam). The color was developed using chemiluminescence using Immobilon Western Chemiluminescent HRP Substrate (Millipore).

### Bacterial two-hybrid assay

To investigate the interaction of YdiU and SodA in vivo, the bacterial adenylate cyclase-based two-hybrid (BACTH) system was used as described previously [24, 28, 29]. Firstly, pKNT25-SodA and pUT18C-YdiU plasmids were constructed and then cotransformed into *E. coli* BTH101. Overnight cultured BTH101 negative controls, positive controls and the re-transformed target strains were inoculated into 100 μg/mL Amp^+^ and 50 μg/mL Kan^+^ double antibiotic LB culture media in a 1:100 ratio to re-activate the seeds at 37 °C with 200 rpm until the OD_600_ reached 0.2. Then, different concentrations of H_2_O_2_ (final concentrations were 0, 2, 4, 6 mM) were added to induce expression for 1 h. Finally, the negative controls, positive controls, and the target strains were centrifuged at 5000 rpm for 2 min, the supernatant was discarded, and only 100–200 µL of bacterial sediment was retained. All the strains were spread onto LB-X-Gal detection plates (containing 100 μg/mL Amp^+^, 50 μg/mL Kan^+^, 0.5 mM IPTG, and 40 μg/mL X-Gal). Three parallels were set up. The results were observed after 40 h of incubation at 30 °C. If there is an interaction between YdiU and SodA, the strains will appear blue. If there is no interaction between YdiU and SodA, the strains will appear white.

### Measurement of β-galactosidase activity

The positive controls, negative controls, and target strains were inoculated into LB culture media containing 100 μg/mL Amp^+^ and 50 μg/mL Kan^+^, and grown at 37 °C with 200 rpm until the OD_600_ reached 0.2. Different concentrations of H_2_O_2_ were added to the target strains, and they were further cultured until the OD_600_ reached 0.5. IPTG was then added to induce β-galactosidase gene expression. After harvesting the cells and lysing them with ultrasound, bacterial proteins were extracted to detect β-galactosidase (β-GAL) activity. Three parallel experiments were set up. Utilizing the principle that β-GAL decomposes p-nitrophenyl-β-D-galactopyranoside to produce p-nitrophenol, which has a maximum absorption peak at 400 nm, the activity of β-GAL was calculated by measuring the rate of increase in absorbance. When using a microplate reader, distilled water was first used to zero the instrument, and then the standard solution was diluted with distilled water to 200, 100, 50, 25, 12.5, 6.25, 0 nmol/mL. A standard curve was established based on the absorbance and concentration of the standard tubes. The change in absorbance (ΔA = A_measured_ – A_control_) of the protein extracted from the re-transformed target strain was then incorporated into the standard curve to calculate the amount of product generated [28].

### Protein cross-linking assay

A desalting column was equilibrated with 10 mL of phosphate-buffered saline (PBS, pH 7.4), and 200 μL each of high-purity YdiU and SodA protein solutions were separately loaded onto the equilibrated column; after the samples completely entered, elution was performed by adding PBS, and the eluate was collected for protein concentration determination using a BCA protein assay kit, followed by adjusting protein concentrations to a molar ratio of YdiU to SodA of 1:3 to form a 10 μL reaction system; when the protein concentration was < 5 mg/mL, bis (sulfosuccinimidyl) suberate (BS3) crosslinker was added to the protein mixture at a molar ratio of 20–50:1 (crosslinker: protein), the system was adjusted to 10 μL with PBS and gently mixed, incubated on ice for 2 h, quenched by adding quenching buffer to a final concentration of 50 mM Tris–HCl (pH 7.5), incubated at room temperature for 15 min, and then subjected to SDS-PAGE analysis.

### In vitro UMPylation assay

In vitro UMPylation assay experiments were constructed using biotin-labeled UTP as the UMP donor (biotin-16-UTP) as described previously [11]. To do this, 4 mg of purified SodA protein was incubated with or without 1 mg YdiU^475^ in a 10 μL reaction buffer (containing25 mM Tris–HCl (pH7.5), 1 mM DTT, 100 mM NaCl, 10 mM MnCl_2_). After incubation at 30 °C for 1 h, the reaction mixture was analyzed by 12% SDS-PAGE and streptavidin-HRP blot was performed as previously described [20, 23, 24].

### Determination of SodA enzyme activity in vitro

In vitro UMPylation assay experiments were performed as described above. The activities of native SodA, 0.1 mM H_2_O_2_ treated SodA and 0.3 mM H_2_O_2_ treated SodA before and after UMPylation in vitro were determined by NBT method (Beijing Solebao Biotechnology Co., LTD. BC0170). According to the kit instructions, the optimal protein concentration for SOD activity detection is 20–80 μg/mL, with the ideal concentration at 40 μg/mL. Thus, purified SodA was uniformly diluted to 40 μg/mL to exclude concentration-dependent interference on enzymatic activity. According to the instructions of the operation procedure, the protein sample and the detection reagent were thoroughly mixed in the 96-well enzyme label plate, and the absorbance at the wavelength of 560 nm was measured after 30 min of water bath at 37 ℃. The absorbance was recorded as A, A control, A1 blank and A2 blank, respectively. The experiment was repeated three times. The calculation formula is as follows:

$${\text{Inhibition percentage }} = \Delta {\text{A blank }} - \Delta {\text{A determination }}/\Delta {\text{A blank }} \times {1}00\%$$

$${\text{SOD activity }} = \left[ {{\text{inhibition percentage }} \div \, \left( {{1} - {\text{ inhibition percentage}}} \right) \, \times {\text{V total}}} \right]/ \, \left( {{\text{V sample }} \times {\mathrm{Cpr}}} \right) \, \times {\mathrm{F}}$$

In the above calculation formula: ΔA blank = A1 blank—A2 blank, ΔA determination = A—A control (V total: total volume of the reaction, Cpr: the concentration of Sample protein, F: sample Dilution).

### Analysis of antioxidant enzyme activities of *Salmonella*

Superoxide dismutase (SOD), catalase (CAT), peroxidase (POD), and thioredoxin peroxidase (TPX) activities were quantified spectrophotometrically using the CheKine™ Micro Assay Kits KTB1030, KTB1042 (ammonium molybdate colorimetric method), KTB1150, and KTB1660, respectively. WT and Δ*ydiU Salmonella* strains were cultured separately in liquid medium containing graded concentrations of hydrogen peroxide (H_2_O_2_; 0, 0.1, 0.3, and 0.5 mM) and incubated at 37 °C with shaking until the optical density at 600 nm (OD_600_) reached 0.5. Cells were harvested by centrifugation (8000 × *g*, 5 min, 4 °C), washed twice with ice-cold phosphate-buffered saline (PBS; pH 7.4) to remove residual medium, and resuspended in the lysis buffer supplied with each assay kit. Lysis was carried out on ice by probe sonication under optimized conditions to ensure complete cell disruption without compromising enzyme integrity. Following a second centrifugation (12 000 × *g*, 10 min, 4 °C), the supernatants were collected as crude enzyme extracts. Enzymatic assays were performed in 96-well microplates according to the manufacturer’s instructions, with incubations maintained at 37 °C for the specified durations. Absorbance was measured at enzyme-specific wavelengths: 450 nm (SOD), 405 nm (CAT), 470 nm (POD), and 340 nm (TPX). Enzyme activities were calculated using the kit-provided formulas and normalized to the number of viable bacterial cells. All experiments were conducted in biological triplicate to ensure statistical robustness and experimental reproducibility.

### In-gel activity staining of SOD

SOD activity was assayed by native polyacrylamide gel electrophoresis (Native-PAGE) followed by in-gel activity staining. Electrophoresis was performed on a discontinuous gel system consisting of a 5% stacking gel and a 10% separating gel, using Tris–glycine buffer (pH 8.3, SDS-free) as the running buffer. The entire electrophoretic run was carried out at a constant voltage under refrigerated conditions (4 °C) to maintain enzymatic activity. After electrophoresis, gels were incubated in the dark in phosphate buffer (0.2 M, pH 7.8) containing 0.48 mM nitroblue tetrazolium (NBT) and 30 µM riboflavin. A subsequent incubation with 0.05% (v/v) TEMED promoted formazan formation, yielding a uniform blue background; SOD-active bands manifested as distinct, unstained zones against this background.

### Identification of UMPylation sites by MS

The UMPylated SodA was analyzed by 12% NuPAGE, stained with Coomassie brilliant blue, and then excised from the gel and digested overnight with trypsin. To confirm the UMPylation sites, digested peptides were desalted with a ZipTip C18 column (Millipore, Billerica, MA) and LC–MS/MS analysis was performed as previously described [23]. Using Mascot software, MS/MS spectra were searched against the database (UniProt no. 83333) of *E. coli* (strain K-12), and UMP-modified amino acids (306.025302, H11C9N2O8P) were searched for Tyr, His, Ser and Thr residues [24].

### Survival rate under H_2_O_2_

For the experiments shown in Figure 1D, *S.* Typhimurium WT and Δ*ydiU* cells were grown to logarithmic phase (OD_600_ ~ 0.4) in LB medium and then H_2_O_2_ was added to the bacterial solution to a final concentration of 0.5 mM. Samples were withdrawn after exposure to 0.5 mM H_2_O_2_ for 3 h, serially diluted into PBS (pH 7.4), and 250 μL samples of 10^6^ dilutions were plated on LB agar plates. Plates were incubated at 37 °C for 24 h and then the colonies were counted. The percentage of bacterial survival was determined (with time zero representing 100%) as a function of the duration of oxidative stress exposure.

### Growth curve under H_2_O_2_ condition

Bacterial growth was monitored using a Bioscreen C Automatic Growth Analyzer (Thermo Electron Corporation) at 37 °C with a honeycomb microplate (Thermo Electron Corporation). Cultures for growth measurements were prepared by gradient dilution of the bacteria into LB medium plus 0.5 mM H_2_O_2_. Into a 96-well plate, 300 μL of diluted culture was added into each well and every strain was assayed in triplicate. Bacterial growth was measured at 600 nm absorbance every 5 min for 24 h.

### Structural analysis

The crystal diffraction data of native SodA and 0.15 mM H_2_O_2_ treated SodA were processed by HKL2000 software, and the results showed that both belonged to P1211 space group. The structures were analyzed by molecular substitution method, and then optimized using the automatic correction of the REFMAC5 program in the CCP4 software package and the manual correction of the Coot software to finally obtain values of Rmerge, Completeness (%), R-work, and R-free that meet the requirements [30].

## Results

### YdiU is involved in the regulation of oxidative stress of *Salmonella*

Our previous data showed that YdiU was expressed at a very low level when *Salmonella* was cultured in Luria–Bertani (LB) medium but expression was strongly induced when H_2_O_2_ was added [20]. H_2_O_2_ is an oxidant that can simulate the effect of oxidative stress on the survival of *Salmonella*. The tested H_2_O_2_ concentrations were determined according to preliminary stress assays of *Salmonella*. These sublethal doses were chosen to trigger moderate oxidative stress without excessive cell damage. To further investigate the correlation between H_2_O_2_ and YdiU expression, the mRNA and protein levels of YdiU were measured for *Salmonella* cultured in LB medium supplemented with different concentrations of H_2_O_2_. Our data showed that the expression of YdiU was efficiently induced by H_2_O_2_, and expression increased with increased H_2_O_2_ concentration (Figure 1A, B). Specifically, when the final concentration of H_2_O_2_ was 0.5 mmol/L, the expression of *ydiU* was the highest, 15 times higher than that of untreated cells (Figure 1A), and protein expression also increased strikingly (Figure 1B, C). These data demonstrate that the oxidative stress signal dramatically induced YdiU expression.

To explore the function of YdiU in oxidative stress, growth and survival rate were monitored using WT and Δ*ydiU Salmonella* grown under 0.5 mmol/L H_2_O_2_ conditions. After adding H_2_O_2_, the growth of WT and Δ*ydiU* was inhibited, but the Δ*ydiU* strain was less affected than the WT strain (Figure 1D). In the survival experiment, the survival rate of the Δ*ydiU* strain was higher than that of WT under the 0.5 mmol/L H_2_O_2_ oxidative stress (Figure 1E).

Consistent with our in vitro oxidative stress phenotypes, the in vivo virulence of the Δ*ydiU* has been characterized in our previous work [23]. In murine infection models, the Δ*ydiU* strain exhibited markedly enhanced pathogenicity relative to the WT strain. This elevated in vivo virulence is mechanistically coupled to the enhanced antioxidant defense of the Δ*ydiU* characterized herein. This adaptive oxidative resistance enables the pathogen to evade host oxidative killing, thereby promoting intracellular survival, tissue colonization, and subsequent virulence development in vivo. To avoid data redundancy and maintain the thematic focus of this manuscript, detailed animal experimental data are not reproduced herein, and readers can refer to our published study for comprehensive in vivo evidence.

### SodA and YdiU interacted under oxidative stress

We previously identified the oxidative stress protein SodA as an UMPylated modified substrate of YdiU [20]. To clarify the physiological function and relative contribution of SodA in response to H_2_O_2_ stress at the whole-cell level. We determined the activities of four SOD isoforms (Sod A, Sod B, Sod CI and CII) in WT and Δ*ydiU Salmonella* under normal culture conditions and after H_2_O_2_ exposure. Total bacterial lysates were separated by native PAGE, and in-gel SOD activity staining was subsequently performed to detect the activity of each SOD isoform [31]. Three major SOD isoforms were separated and distinguished according to their differential electrophoretic mobility in native gels. From the cathode to the anode (top to bottom of the gel), these bands were identified as SodA (Mn‑SOD) and SodB (Fe‑SOD), followed by a merged band containing both SodCI and SodCII (Cu/Zn‑SOD isoforms). Owing to their highly similar molecular weight and isoelectric properties under non‑denaturing conditions, SodCI and SodCII could not be fully separated in the current native‑PAGE system and therefore co‑migrated as a single activity band. Interestingly, the SodA band displayed the strongest activity signal among all detected SOD isoforms. In WT strain, SodA activity decreased gradually with the increase in H_2_O_2_ concentration. In contrast, the Δ*ydiU* exhibited a marked elevation of SodA activity under escalating H_2_O_2_ stress (Additional file 4). Collectively, these findings indicate that SodA acts as a crucial antioxidant enzyme in response to oxidative stress in *Salmonella* Typhimurium, and YdiU negatively modulates SodA activity under peroxide-induced oxidative conditions.

**Figure 2:**
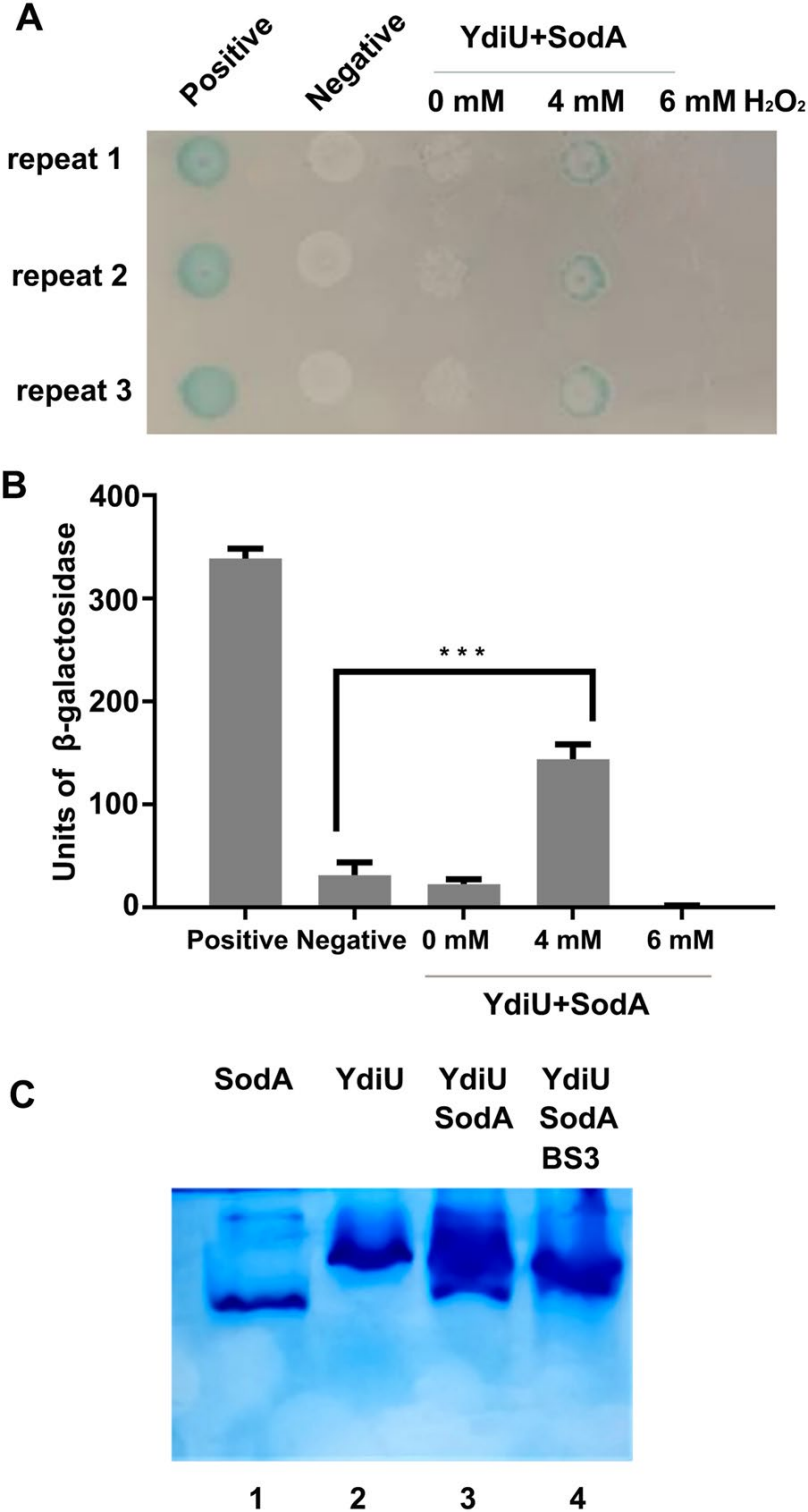
**SodA and YdiU interacted under H**_**2**_**O**_**2**_ **pressure**. **A** Direct interaction between YdiU and SodA was detected by a bacterial two-hybrid method under 0, 4 and 6 mM H_2_O_2_ stress, respectively. **B** Corresponding strains were tested by β-galactosidase assays in LB-X-Gal plates, which displayed above the histogram. The values represent the mean ± standard deviation of three repeated results and statistical significance is indicated by *** *P* < 0.001 as compared with negative control using a *t* test. **C** BS3 cross-linking assay to analyze the interaction between SodA and YdiU in vitro. Purified proteins were subjected to BS3 cross-linking and analyzed by native-PAGE with Coomassie blue staining. Lane 1: SodA alone; Lane 2: YdiU alone; Lane 3: SodA + YdiU without cross-linker; Lane 4: SodA + YdiU with BS3 cross-linker. The formation of higher molecular weight cross-linked complexes (Lane 4) indicates the direct interaction between SodA and YdiU.

However, how *Salmonella* regulates SodA by UMPylation to deal with oxidative stress has not been fully elucidated. To determine whether there is an interaction between YdiU and SodA, we constructed a bacterial two-hybrid system based on adenylate cyclase reconstitution. Two plasmids with a T18 subunit (18 kDa fragment containing the calmodulin binding site) and a T25 subunit (25 kDa fragment with the catalytic site) were recombined with foreign genes *ydiU* and *sodA*, respectively. We confirmed pUT18C-*ydiU* and pKNT25-*sodA* by electrophoresis and sequencing (Additional file 5A). Interaction of the two target proteins in the cell, in the presence of calmodulin, can promote the close contact of the T18 and T25 subunits and increase the level of intracellular cAMP. This higher level of cAMP activates the expression of lactose and maltose operons, allowing screening for blue colonies on LB-X-Gal plates [28]. The strong interaction between T18-zip and T25-zip plasmids was used as a positive control and the empty T18 and T25 plasmids were used as negative controls. Interesting, an interaction between YdiU and SodA was not observed on LB-X-Gal plates under the initial testing conditions (Additional file 5B). We wondered if the interaction might depend on the concentration of H_2_O_2_, as SodA actively protects *Salmonella* in response to oxidative stress. Our preliminary screening revealed that YdiU and SodA do not interact under low H_2_O_2_ stress, and their binding relies on moderate oxidative stimulation. Moreover, lethal high-dose H_2_O_2_ would damage proteins and destabilize the reporter system. Thus, the applied concentrations were optimized to trigger specific protein interaction without causing fatal cytotoxicity. To test this, we next used 2, 4 and 6 mM H_2_O_2_ to simulate oxidative stress and repeated the experiment. The results showed that when at 2 mM and 4 mM H_2_O_2_, the colonies were light blue, but when the concentration of H_2_O_2_ was too high (6 mM H_2_O_2_), *Salmonella* did not form colonies (Figure 2A and Additional file  5C). The strength of protein–protein interaction was quantified by measuring β-galactosidase (β-GAL) activity [29]. The result indicated that there is a certain interaction between SodA and YdiU under H_2_O_2_ pressure (Figure 2B).

To investigate the direct interaction between SodA and YdiU in vitro, we performed a BS3-mediated chemical cross-linking assay. As shown in Figure 2C, when the purified proteins were analyzed by native-PAGE and Coomassie blue staining: Lane 1 (SodA alone) showed two distinct bands corresponding to the monomeric and native dimeric forms of SodA. Lane 2 (YdiU alone) displayed a single band at the expected molecular weight of YdiU. In the absence of BS3 (Lane 3), co-incubation of SodA and YdiU did not produce new bands, indicating no stable complex formation under native-PAGE conditions. In contrast, treatment with the cross-linker BS3 (Lane 4) resulted in the appearance of a prominent band that was absent in the control groups. This new band corresponds to the covalently cross-linked SodA-YdiU complex, confirming that SodA and YdiU directly interact with each other in vitro.

### YdiU UMPylates SodA under oxidative stress

**Figure 3:**
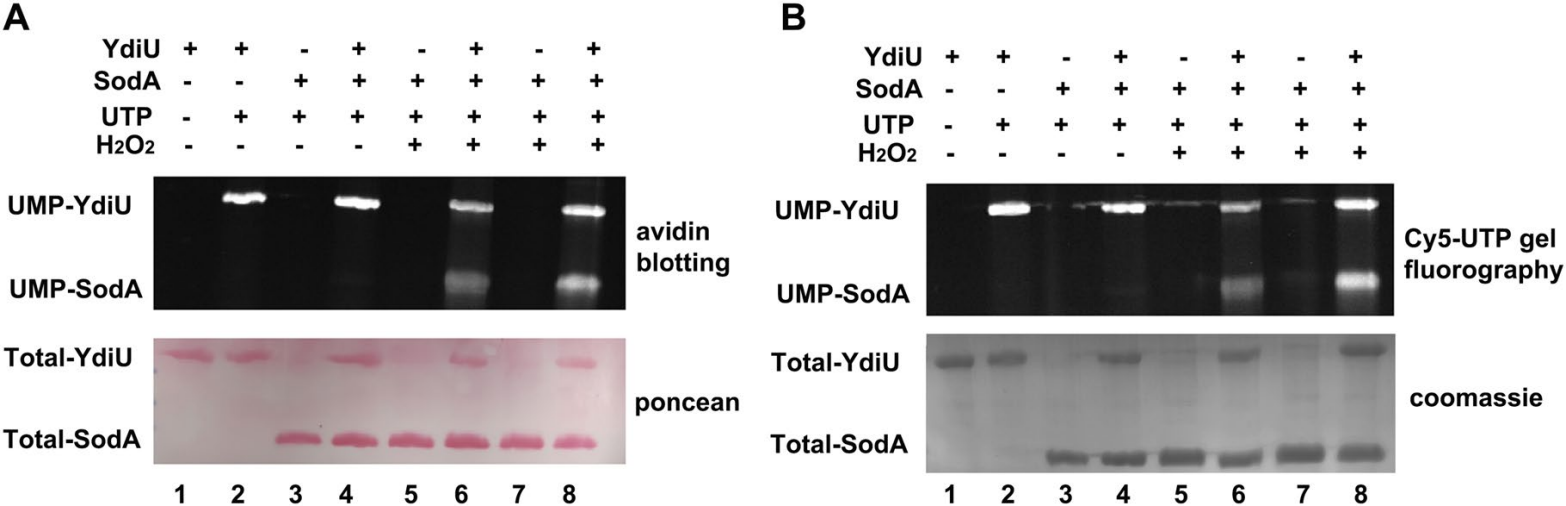
**YdiU UMPylates SodA under oxidative stress**. **A** UMPylation of native SodA and H_2_O_2_ treated SodA. **B** The SodA was obtained from *ydiU* mutant of *E. coli*. UMPylation of native SodA and H_2_O_2_ treated SodA. Lane 1: YdiU control; Lane 2: YdiU auto-UMPylation; Lane 3: native SodA auto-UMPylation; Lane 4: YdiU-mediated UMPylation of native SodA; Lane 5: SodA treated with 0.1 mM H_2_O_2_ alone; Lane 6: YdiU-mediated UMPylation of 0.1 mM H_2_O_2_-treated SodA; Lane 7: SodA treated with 0.5 mM H_2_O_2_ alone; Lane 8: YdiU-mediated UMPylation of 0.5 mM H_2_O_2_-treated SodA. To do this, 4 mg of purified SodA protein was incubated with or without 1 mg YdiU in a 10 μL reaction buffer. The “-” and “ + ” of YdiU indicate whether exogenous YdiU is added to the reaction system (“-”: no exogenous YdiU added; “+”: exogenous YdiU added).

The above results indicate that SodA and YdiU can interact under oxidative stress. Next, we explored the regulatory effects of YdiU on SodA expression at transcriptional and translational levels. To do this, qRT-PCR and western blotting were used to evaluate the transcription levels and protein levels. The results clearly showed that the mRNA level and protein level of SodA did not significantly change under low concentration of H_2_O_2_ between WT and Δ*ydiU Salmonella*, but there was a slight increase under high concentration H_2_O_2_ between WT and Δ*ydiU* (Additional file  6A, B). These data indicate that YdiU modulates SodA function at the post-translational level, independent of transcriptional and translational regulation. Previous studies in our laboratory have shown that YdiU can UMPylate itself and its substrate [20]. We next tested whether SodA can be UMPylated by YdiU. We extracted and purified the SodA protein under native and oxidative stress (SodA purified from conditions of oxidation pressure was dialyzed to remove residual H_2_O_2_ to prevent effects on subsequent experiments). SodA substrate protein, YdiU protein, and biotin-labeled UTP were mixed, and the UMPylated substrate protein was identified by western blot. As shown in Figure 3A, native SodA displayed low susceptibility to YdiU-mediated UMPylation, whereas SodA exposed to H_2_O_2_ stress was readily UMPylated by YdiU. To exclude potential interference from endogenous *E. coli* YdiU, SodA was expressed in an *ydiU*-deficient mutant strain of *E. coli*, and subsequent UMPylation assays were further performed in vitro. Consistent with the above results, in vitro detection (Figure 3B) confirmed that unstressed SodA remained barely UMPylated, while oxidatively stressed SodA was efficiently modified by YdiU. Endogenous YdiU in *E. coli* exerted negligible regulatory effects on SodA, which is consistent with its previously reported physiological role in bacterial stress adaptation [20]. Collectively, these findings demonstrate that YdiU-dependent regulation of SodA occurs via protein post-translational UMPylation modification.

### Oxidation pressure influence the secondary structure of SodA

**Figure 4:**
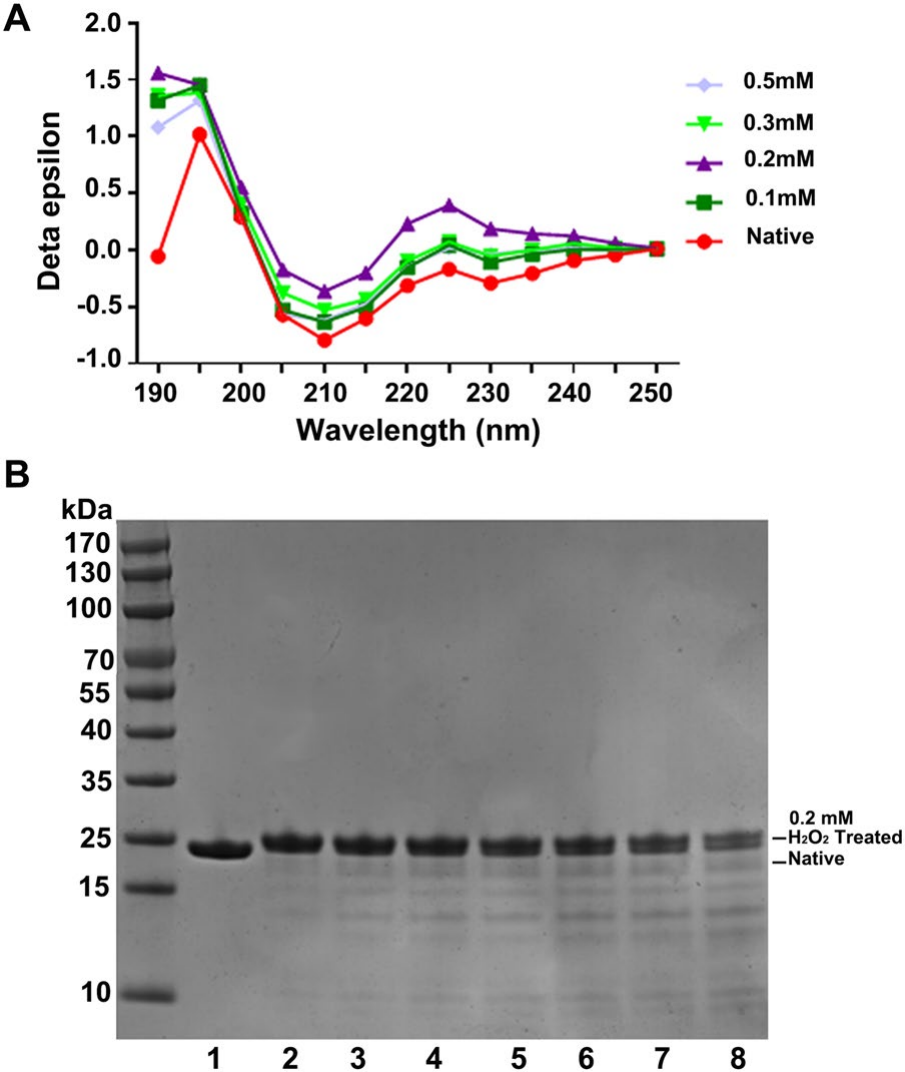
**Oxidation pressure influence the secondary structure of SodA**. **A** The circular dichroism spectrometer of British Applied Photophysics Company is used to analyze the difference of secondary structure of SodA under different concentration H_2_O_2_ (0, 0.1, 0.2, 0.3 and 0.5 mM H_2_O_2_). **B** 12% SDS-PAGE of SodA protein before and after oxidative stress. 1 refers to native SodA, 2–8 refers to SodA that reacts for 1, 2, 3, 4, 6, 9, 18 h of 0.2 mM H_2_O_2_. The main bands are the SodA protein; the weak bands are the degradation products of the target proteins, which are caused by slight protein degradation during the reaction process. All experiments in this study were repeated at least three times independently (*n* ≥ 3), and the images shown in the manuscript are representative of the most typical results of these repeated experiments.

In order to explore the differences between SodA purified from native or H_2_O_2_-treated cells and to analyze how UMPylation affects the structure of SodA, we used circular dichroism (CD) and gel filtration chromatography. The circular dichroism spectra were analyzed for native SodA and SodA under0, 0.1, 0.2, 0.3 and 0.5 mM H_2_O_2_ pressure and the results showed that H_2_O_2_ pressure had obvious influence on the secondary structure of SodA (Figure 4A). Among them, the secondary structure changes were the most significant when treated with 0.2 mM H_2_O_2_, while they were not obvious when treated with 0.5 mM H_2_O_2_. We speculate 0.2 mM H_2_O_2_ is the critical concentration for inducing the conformational change of SodA protein, and a higher concentration (≥ 0.5 mM) will cause irreversible denaturation of SodA protein, making it impossible to detect the subtle conformational changes caused by UMPylation. Therefore, we treated the SodA protein with 0.2 mM H_2_O_2_ at different time intervals. Electrophoretic results showed slight changes between native SodA and H_2_O_2_-treated SodA (Figure 4B).

### Crystal structures of native SodA and 0.15 mM H_2_O_2_ treated SodA

CD analysis revealed that oxidative stress greatly altered the secondary structure of SodA, with remarkable structural changes occurring at 0.1–0.2 mM H_2_O_2_. Thus, a moderate concentration of 0.15 mM H_2_O_2_ was chosen to prepare native and oxidatively stressed SodA samples for subsequent structural comparison. We detected crystal growth for the three protein preparations in Index™-HR2-144, with similar crystal shapes (Additional file 7A). Diffraction data were obtained for the three proteins, but only the diffraction data for the native and 0.15 mM treated proteins met the requirements of structural analysis (Additional file 7B).

**Figure 5:**
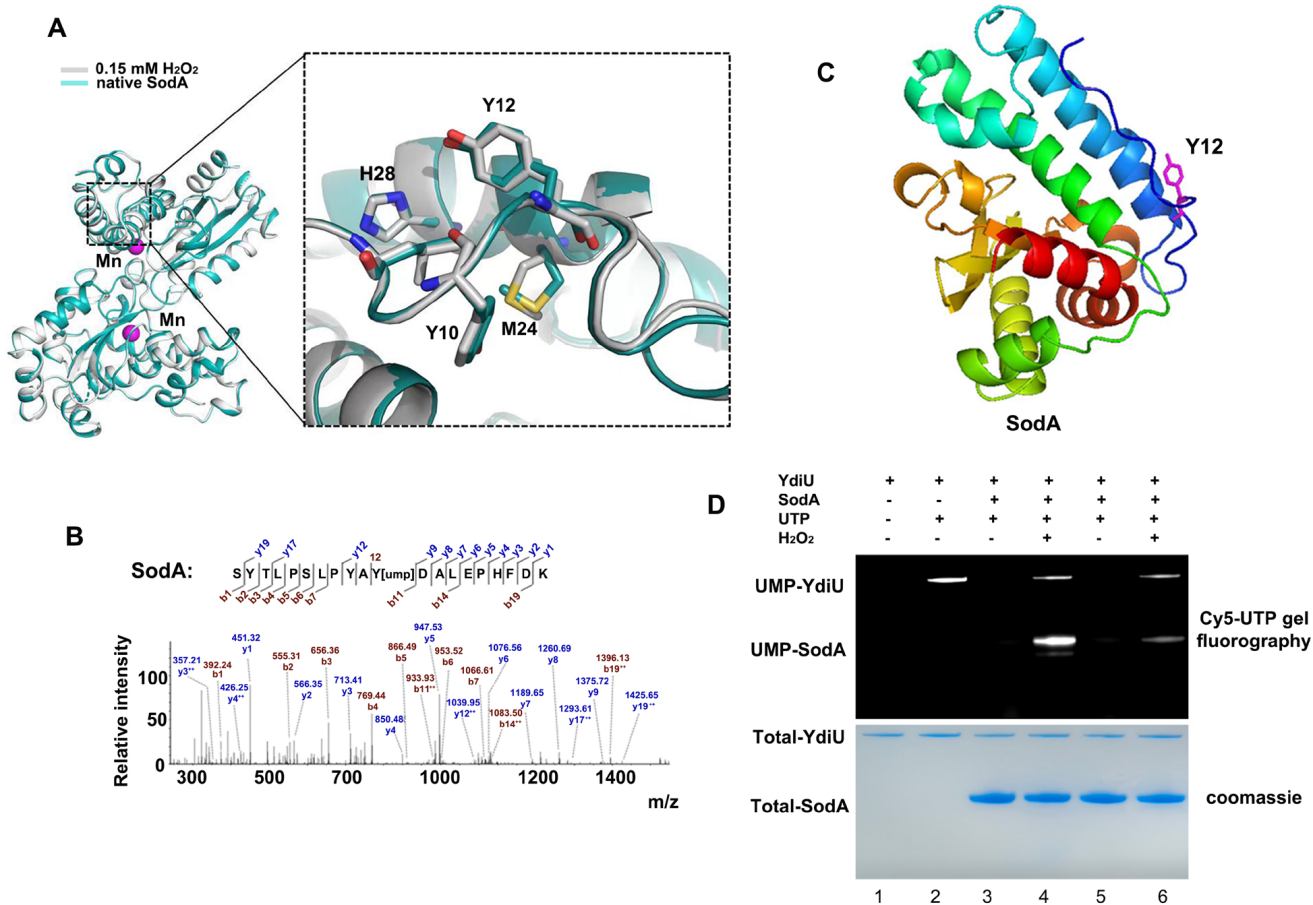
**The structural and UMPylated sites of SodA**. **A** Structural comparison between native SodA and 0.15 mM H_2_O_2_ treated. Pink balls represent manganese ions. The overall structure of SodA did not change greatly under oxidation pressure. The Met24 of SodA under H_2_O_2_ pressure had obvious structural phase migration. **B** Electrospray ionization tandem mass spectrometry (MS/MS) spectra of UMPylated peptides identified in SodA^UMP^. The b and y ions are indicated along the peptide sequence above the spectra. Marking of the UMPylated site of SodA. **C** The Tyr12 of SodA was the UMPylated site. b ions and y ions are ions formed after the peptide is broken at the peptide bond, and they contain part of the amino acids of the peptide. The b ion contains amino acids at the N-terminal of the polypeptide, while the y ion contains amino acids at the C-terminal of the polypeptide. Unique ions (305.1 Da) corresponding to the neutral loss of the UMP group were detected in the MS/MS spectra of UMPylated peptides. **D** In vitro UMPylation of SodA by YdiU under oxidative stress conditions. The UMPylation assay was performed using Cy5-UTP as the substrate. All samples were separated by SDS-PAGE, followed by in-gel fluorescence scanning (top panel) to detect Cy5-labeled UMPylated proteins, and Coomassie blue staining (bottom panel) to verify equal protein loading. Lane 1: YdiU alone (negative control); Lane 2: YdiU + Cy5-UTP (positive control for YdiU auto-UMPylation); Lane 3: Wild-type SodA + YdiU (without H_2_O_2_); Lane 4: Wild-type SodA + YdiU + Cy5-UTP + H_2_O_2_; Lane 5: SodA^H12M^ mutant + YdiU + Cy5-UTP; Lane 6: SodA^H12M^ mutant + YdiU + Cy5-UTP + H_2_O_2_.

The crystal structures of native SodA (PDB: 8KB3, Additional file 7C) and 0.15 mM H_2_O_2_ treated SodA (Additional file 7D) show little change in the overall structure under oxidation pressure. However, there was local conformational shifts for Met24 of SodA under H_2_O_2_ pressure. This residue is positioned head-to-head with Tyr12, suggesting a change the after oxidation (Figure 5A). The UMPylation site of SodA under H_2_O_2_ pressure was identified by electrospray ionization tandem mass spectrometry (LC–MS/MS). The results of mass spectrometry show that Tyr10 and Tyr12 may be modification sites (Figure 5B). So we hypothesized that Tyr12 may be the UMPylated site, and this residue was mapped onto the structure of SodA using PyMOL software (Figure 5C). In the structure of the SodA purified from H_2_O_2_-treated cells, the hydroxyl group of the SodA UMPylated site was exposed, making it more likely to be modified by YdiU. To validate this hypothesis, SodA Tyr12 was mutated to Met12. After purification, in vitro modification assay revealed that UMPylation of the mutant protein was attenuated, confirming Tyr12 as the UMPylation site of SodA (Figure 5D).

### YdiU regulates oxidative stress through negative regulation of SodA activity

**Figure 6:**
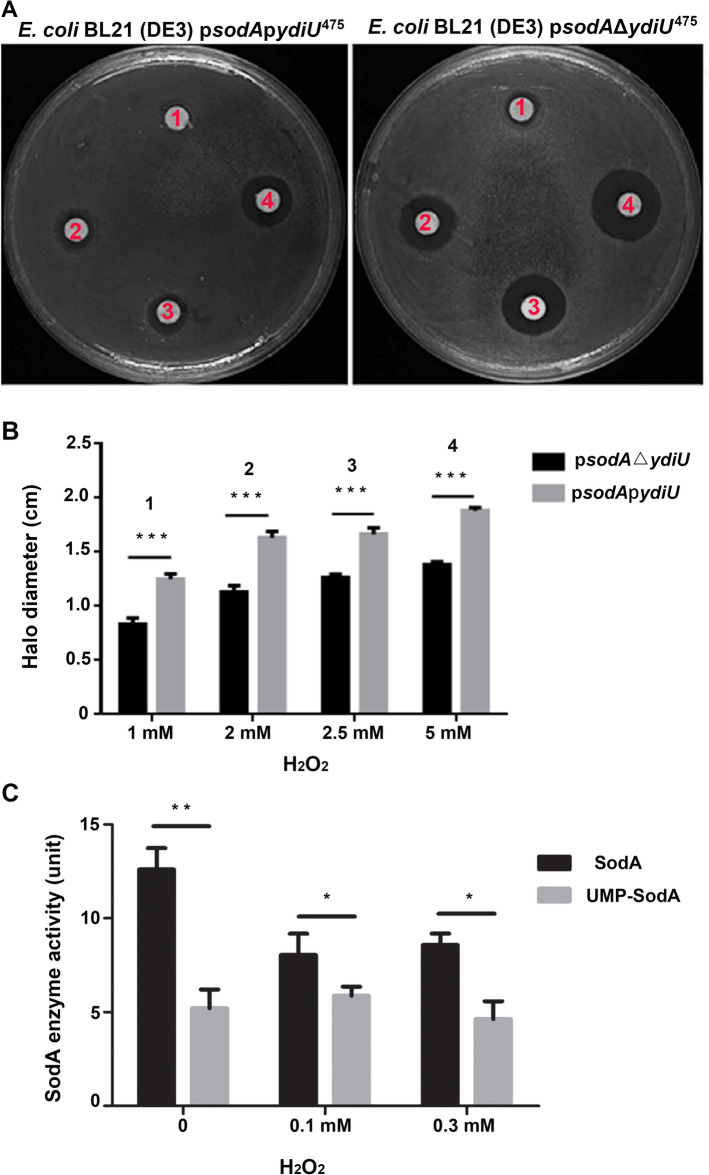
**YdiU negatively regulate SodA activity**. **A**, **B** Disc diffusion assays of *E. coli* BL21 (DE3) p*sodA*p*ydiU*^*475*^ and *E. coli* BL21 (DE3) p*sodA*Δ*ydiU*^*475*^. The numbers (1–4) on the filter discs represent different concentrations of H_2_O_2_, and the images on the below correspond to the above. After overnight exposure, the diameters of the death zones surrounding the H_2_O_2_-soaked filters were measured. **C** SodA enzyme activities (native SodA, 0.1 mM H_2_O_2_ treated SodA and 0.3 mM H_2_O_2_ treated SodA) before and after UMPylation were determined by NBT method. The data are the means ± SE of three independent experiments.

The effect of YdiU on SodA activity under oxidative pressure was next analyzed using co-expression strains of BL21 containing SodA/pGL01 and the empty vector pET29b or SodA/pGL01 and YdiU^475^/pET29b co-transformed plasmids. In a disk diffusion assay, the H_2_O_2_ adsorbed on a filter plate cause oxidative stress in *E. coli*. In this assay, if the SodA enzyme activity is high, the inhibition zone is smaller, and if activity is low, the zone is larger. The results showed that under the different concentration of H_2_O_2_ stress, the inhibition zones of cells expressing SodA and YdiU^475^ were larger than those of cells expressing only SodA and not YdiU^475^ (Figure 6A, B). In addition, SodA enzyme activity before and after UMPylation in vitro were determined by NBT method. As shown in the Figure 6C, the enzyme activity of SodA (native SodA, 0.1 mM H_2_O_2_ treated SodA and 0.3 mM H_2_O_2_ treated SodA) after UMPylation decreased significantly. In particular, the activity of native SodA enzyme activity decreased by 2.5 times after UMPylation. However, after the mutation, the enzymatic activity before and after the modification remained unchanged (Additional file 8). These results suggested that YdiU negatively regulates the enzyme activity of SodA.

### YdiU modulates SodA activity but not catalases and peroxidases under oxidative stress

**Figure 7:**
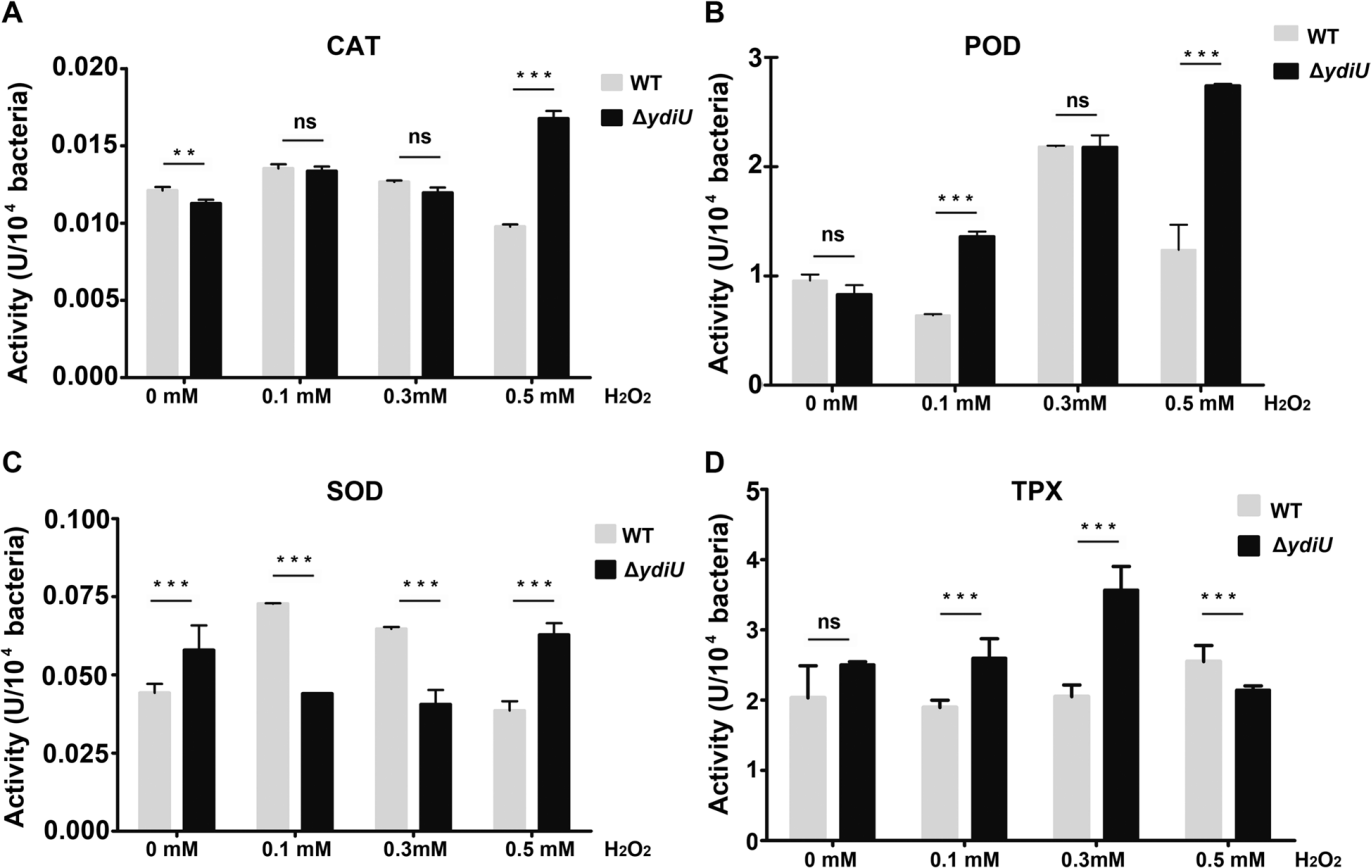
**Antioxidant enzyme activities of WT and Δ*ydiU Salmonella*** **Typhimurium upon gradient H**_**2**_**O**_**2**_** exposure**. Activities of CAT (**A**), POD (**B**), SOD (**C**) and TpX (**D**) in strains under 0, 0.1, 0.3, 0.5 mM H_2_O_2_ stress. All data are presented as the mean ± standard deviation (SD) of three independent biological replicates. Statistical significance was determined via two-way ANOVA: **p < 0.01, ***p < 0.001; ns, no significant difference.

Catalases and peroxidases are core antioxidant defenders against hydrogen peroxide-mediated oxidative damage, functioning coordinately with SOD to maintain intracellular redox homeostasis. To further explore the antioxidant regulatory network governed by YdiU, we subsequently measured the activities of major catalase isoforms (KatG, KatE, KatN), peroxidase/peroxiredoxin proteins (AhpC/TPX), total peroxidase (POD), and total SOD in WT and Δ*ydiU* mutant *Salmonella *Typhimurium. All determinations were conducted under routine culture conditions and gradient H_2_O_2_ stress (0, 0.1, 0.3, and 0.5 mM ), and the relevant activity data are presented in Figure 7. In the WT strain, H_2_O_2_ induction upregulated YdiU, which decreased SOD activity and subsequently reduced H_2_O_2_ production, resulting in lower activities of CAT, POD, and TPX. In the Δ*ydiU* mutant, without YdiU-mediated modification of SodA, SOD activity was increased, leading to higher intracellular H_2_O_2_ levels and consequently elevated activities of CAT, POD, and TPX. Together, these results support that YdiU specifically regulates the antioxidant network by targeting SodA activity.

## Discussion

Mammals, plants, and *E. coli* encode YdiU homologs (SelO in mammals/plants) with conserved redox regulatory roles [32–35], leading us to hypothesize a general role of YdiU/SelO in cellular redox regulation. Here, H_2_O_2_ dramatically induced *ydiU* expression, and Δ*ydiU* exhibited stronger antioxidant capacity in vitro (Figure 1), demonstrating YdiU’s importance in *Salmonella* oxidative stress regulation. While YdiU is induced by ROS, it does not directly resist ROS but maintains redox balance via UMPylation of ROS-scavenging enzymes (e.g., SodA); its exact regulatory mechanism remains unclear.

Bacterial two-hybrid assays showed SodA-YdiU interaction under H_2_O_2_ stress (Figure 2), and YdiU-mediated SodA UMPylation was significantly enhanced under this condition (Figure 3A). Combined with native PAGE in-gel SOD activity staining, our results confirm SodA is essential for *Salmonella* oxidative stress resistance, with YdiU acting as a negative regulator of SodA activity (Additional file 4). Under oxidative stress, YdiU may regulate *Salmonella* antioxidant capacity via SodA UMPylation—likely a more energy-efficient and rapid mechanism than direct protein–protein interaction. Mass spectrometry identified Tyr12 as the SodA UMPylation site (Figure 3B, C); further investigation of the relationship between this site and SodA structural changes under H_2_O_2_ stress will clarify the molecular mechanism of YdiU-mediated SodA modification.

Redox sensing molecular mechanisms depend on ROS/RNS-induced protein conformational changes [36]. H_2_O_2_, a potent oxidant, reacts directly with thiols but slowly with most protein cysteine residues due to high activation energy [37]. During macrophage phagocytosis, *Salmonella* Typhimurium faces steady-state H_2_O_2_ (1–4 μM) and transient bursts (1–10 mM); here, 4–6 mM H_2_O_2_ was used to simulate extreme host oxidative stress and verify antioxidant limits. While debate exists whether proteins are oxidized directly by H_2_O_2_ or via redox carriers, reversible/irreversible protein modifications translate ROS into dynamic biological responses [38].

Circular dichroism (CD) and SDS-PAGE showed significant SodA secondary structure changes and slight protein differences after H_2_O_2_-induced oxidative stress (Figure 4A, B). H_2_O_2_ may induce these changes by oxidizing cysteine sulfhydryl groups (-SH) to disulfide bonds (–S–S–) or interacting with SodA’s active site (e.g., manganese-binding site), with the underlying mechanism to be explored. While CD reflects overall protein structure, X-ray crystal analysis provides atomic-resolution details [39, 40]. We observed H_2_O_2_-induced conformational migration of SodA Met24 (adjacent to Tyr12, Figure 5A), which may cause altered PAGE migration (Figure 4A) and expose Tyr12 (the UMPylation site) for YdiU modification. This study provides structural support for investigating YdiU’s role in *Salmonella* oxidative stress response.

Most SOD activity regulation studies focus on transcriptional/translational changes, but recent work highlights post-translational modifications (PTMs), such as MnSOD nitration [41] and acetylation [42], which alter activity. We found YdiU negatively regulates SodA activity: *ydiU* deletion abolishes SodA UMPylation, causing its abnormal activation. Contrary to intuition, excessive SodA activity is not always beneficial for *Salmonella*, and this negative regulation reflects a sophisticated survival strategy. First, excessive SodA disrupts intracellular O_2_⁻/H_2_O_2_ balance, leading to H_2_O_2_ accumulation and oxidative damage. Second, as a virulent factor, overly active SodA may overactivate the host immune response [43]. Third, in *E. coli*, single deletion of *sodA* or *sodB* only slightly enhances cellular susceptibility to paraquat (methyl viologen), a potent oxidant that generates intracellular superoxide anions (O_2_⁻), while the *sodA sodB* double mutant exhibits markedly higher sensitivity than the wild-type strain [44]. Fourthly, in *E. coli*, *sodA* expression is subject to regulation by six regulatory proteins, OxyR, SoxR, SoxS, ArcA, ArcB and Fur. Among them, OxyR is a key regulator of the H_2_O_2_ stress response, SoxR/SoxS regulates the superoxide stress response, ArcA/ArcB regulates the redox balance under anaerobic conditions, and Fur regulates the iron metabolism and oxidative stress response [45], and likely similarly in *Salmonella* [46], making YdiU’s negative regulation just one aspect of a complex network. Overall, YdiU-mediated SodA downregulation optimizes *Salmonella* survival by avoiding excessive host immune activation and ensuring adaptability to oxidative stress, embodying its evolutionary adaptability to complex environments.

**Figure 8:**
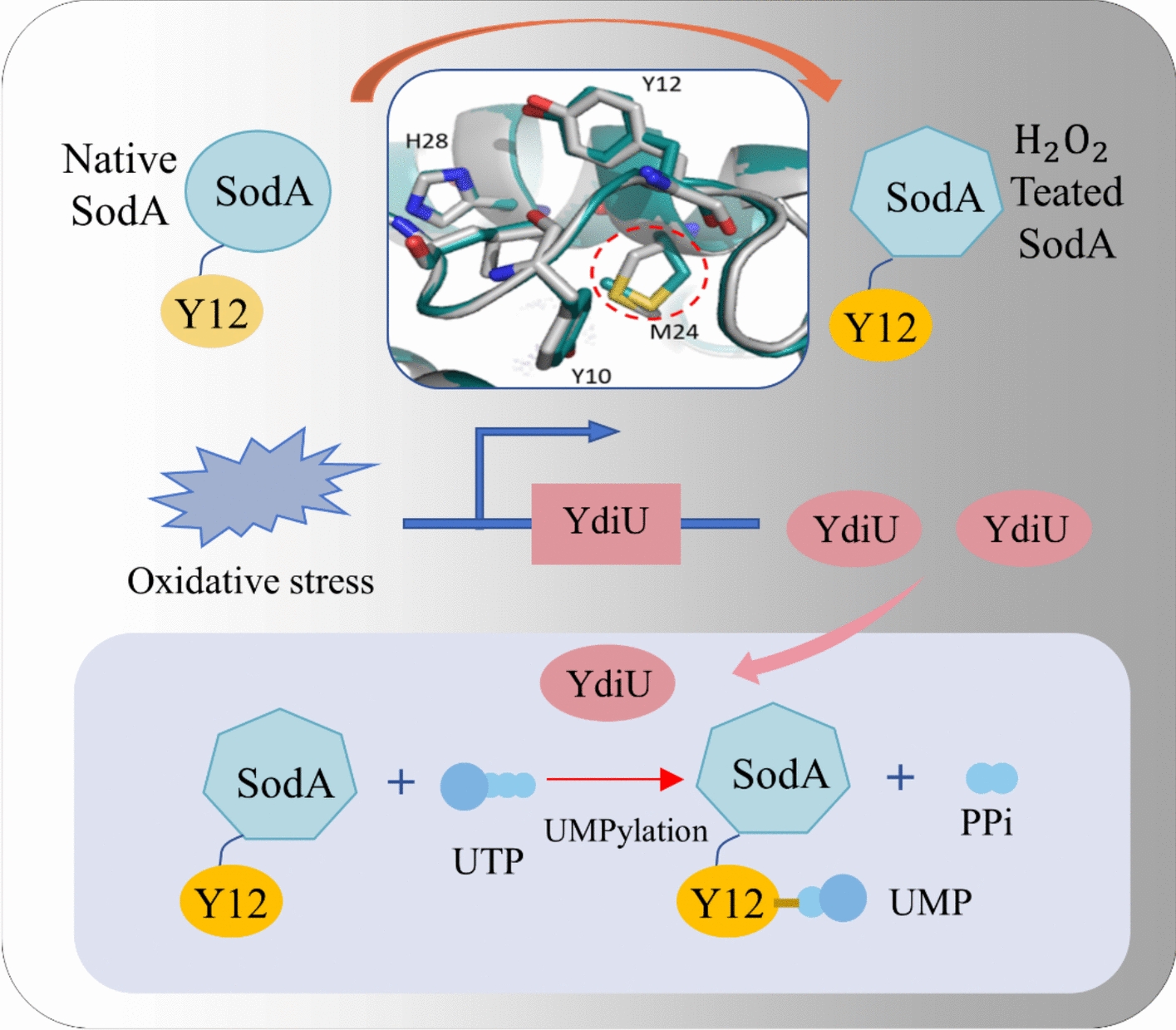
**Molecular mechanism model of YdiU regulates oxidative stress of** ***Salmonella*** **via UMPylation of SodA**. After *Salmonella* enters host cells, YdiU is expressed and SodA under oxidative stress could be UMPylated by YdiU, and the Tyr12 of SodA was the UMPylated site. The Met24 of SodA under H_2_O_2_ pressure had obvious structural phase migration. It was head-to-head with Tyr12, which affects the conformation of the UMPylated site after oxidation. As a result, the hydroxyl group of the SodA UMPylated site was exposed, making it more likely to be modified by YdiU.

Based on these results, we constructed a model of YdiU-mediated SodA UMPylation in intracellular *Salmonella*. Upon host cell entry, oxidative stress upregulates YdiU expression and induces SodA secondary structure changes. H_2_O_2_-treated SodA undergoes UMPylation by YdiU at Tyr12; H_2_O_2_ also induces conformational migration of SodA Met24 (adjacent to Tyr12), exposing Tyr12’s hydroxyl group to facilitate YdiU modification, ultimately enabling YdiU to negatively regulate SodA activity (Figure 8). This study provides strong experimental support for understanding *Salmonella*’s oxidative stress adaptation in host cells.

UMPylation, a reversible, rapid and flexible post-translational modification, regulates enzyme activity quickly in response to stress (e.g., ROS) without de novo protein synthesis—more suitable for bacterial adaptation to acute oxidative stress than transcriptional regulation. Energy-dependent (requiring UTP), UMPylation is triggered by intracellular redox changes (e.g., H_2_O_2_ accumulation) that activate UMPylating enzymes like YdiU. YdiU can UMPylate other metabolic and stress-related proteins, which we will explore further. YdiU-mediated SodA UMPylation avoids excessive H_2_O_2_ accumulation (SodA’s product) to reduce oxidative damage and allows catalases to dominate H_2_O_2_ degradation, forming a coordinated network with SodA to maintain intracellular redox balance [45, 46].

## Supplementary Information

**Additional file 1 Strains used in this study.**

**Additional file 2 Primers used in this study.**

**Additional file 3 Plasmids used in this study.**

**Additional file 4 In-gel SOD isoform activity profiling in WT and Δ*ydiU S*. Typhimurium under H₂O₂-induced oxidative stress.** SOD enzymatic activities were resolved by native polyacrylamide gel electrophoresis (Native-PAGE) followed by nitroblue tetrazolium (NBT)-riboflavin-based in-gel activity staining. Strains examined included the WT *S*. Typhimurium and an isogenic ΔydiU deletion mutant. Bacterial cells were grown to an optical density of 0.5 at 600 nm (OD₆₀₀) in medium containing 0, 0.1, 0.3, or 0.5 mM H_2_O_2_, and subsequently harvested and lysed. Three distinct, reproducible SOD activity bands were observed, ordered from highest to lowest electrophoretic mobility: SodA (Mn-SOD), SodB (Fe-SOD), and the co-migrating Cu/Zn-SOD isoforms SodCI and SodCII.

**Additional file 5 The interaction between YdiU and SodA.** (**A**) Identification of positive recombinant strains pUT18C-ydiU, pKNT25-sodA by DNA electrophoresis. (**B**) Direct interaction between YdiU and SodA was detected by a bacterial two-hybrid method. Positive control: recombinant strain BTH101 containing T18-zip and T25-zip vectors. Negative control: recombinant strain BTH101 containing the empty T18 and T25 vectors. 1 and 2 represented the interaction betweenYdiU and SodA under normal circumstances and 2 mM H_2_O_2_ oxidative stress, respectively. (**C**) Direct interaction between YdiU and SodA was detected by a bacterial two-hybrid method under 2, 4 and 6 mM H_2_O_2_ stress, respectively.

**Additional file 6 The expression level of SodA with different concentrations of H**_**2**_**O**_**2**_. (A and B) The transcription and protein levels of SodA in Salmonella cultivated with different concentrations of H_2_O_2 _were detected with qRT-PCR and western blotting, respectively. GapA (also known as glyceraldehyde-3-phosphate dehydrogenase [GAPDH]) was used as a loading control. Lane 1-4: WT, Lane 5-8: Δ*ydiU*.

**Additional file 7 Crystal structure analysis of native SodA and 0.15 mM H**_**2**_**O**_**2**_ treated SodA. (A) The crystal shapes of the native SodA and 0.15 mM H_2_O_2_ treated SodA were similar. (B) Protein diffraction data were received in Shanghai Light Source, and the resolution of native SodA and 0.15 mM H_2_O_2_ treated SodA was 1.8 Å, 2.8 Å, respectively. (C) Crystal structures of native SodA. (D) Crystal structures of 0.15 mM H_2_O_2_ treated SodA.

Supplementary Material 8. **Additional file 8 Impact of UMPylation on the Enzymatic Activity of native SodA and the H12M variant.** Enzymatic activities of native SodA and its H12M point mutant were quantified under both unmodified and UMPylated conditions. Data are presented as mean ± standard deviation (*n *= 3 independent experiments). Statistical significance was determined using unpaired two-tailed Student’s *t*-test. ***p < 0.001; ns, no significant difference (*p* ≥ 0.05).

## Data Availability

No datasets were generated or analysed during the current study.
