# Supplementary material for: Oxidative stress-triggered UMPylation of SodA by YdiU modulates oxidative stress resistance in Salmonella
Source: Vet Res. 2026 Jul 11;57:131. doi: 10.1186/s13567-026-01818-7 (PMC13355359; doi:10.1186/s13567-026-01818-7)
Supplement: Supplementary file 1 — Additional file 1 Strains used in this study. [file 13567_2026_1818_MOESM1_ESM.docx]

**Strains used in this study**

| No | Strains | Relevant characteristic(s) | Source |
| --- | --- | --- | --- |
| 1 | WT *Salmonella* | *Salmonella enterica* serovar Typhimurium ATCC14028, no resistance | American Type CultureCollection |
| 2 | *Salmonella* Δ*ydiU* | *ydiU* knockout strain, no resistance | [20] |
| 3 | *E. coli* BL21(DE3) | T7 expression host, no resistance | Takara Bio Inc. |
| 4 | *E. coli* BL21(DE3)p*sodA* | *sodA*/ pGL01, Amp^+^ | This study |
| 5 | *E. coli* BL21(DE3)p*ydiU* | *ydiU*^475^/ pGL01, Amp^+^ | [20] |
| 6 | *E. coli* BL21(DE3)p*sodA* p*ydiU* | *sodA*/ pGL01, Amp^+^  YdiU^475^/ pET29b (no tag), Kan^+^ | This study |
| 7 | *E. coli* BL21(DE3) p*sodA*Δ*ydiU* | *sodA*/ pGL01, Amp^+^  pET29b (no tag), Kan^+^ | This study |
| 8 | *E. coli* BTH101 | no resistance , strain used for bacterial two-hybrid assay | [23] |
| 9 | *E. coli* BTH101-PC | zip/pKNT25, Kan^+^; zip/pUT18C, Amp^+^, Positive control for bacterial two-hybrid assay | [23] |
| 10 | *E. coli* BTH101-NC | Vector pKNT25 control Kan^+^, Vector pUT18C control Amp^+^, Negative control for bacterial two-hybrid assay | [23] |
| 11 | *E. coli* BTH101-*sodA*-*ydiU* | *sodA*/pKNT25, Kan^+^; *ydiU*/pUT18C, Amp^+^ | This study |
