# Supplementary material for: Oxidative stress-triggered UMPylation of SodA by YdiU modulates oxidative stress resistance in Salmonella
Source: Vet Res. 2026 Jul 11;57:131. doi: 10.1186/s13567-026-01818-7 (PMC13355359; doi:10.1186/s13567-026-01818-7)
Supplement: Supplementary file 2 — Additional file 2 Primers used in this study. [file 13567_2026_1818_MOESM2_ESM.docx]

**Primers used in this study**

| No | Oligonucleotide | Sequence | Application |
| --- | --- | --- | --- |
| 1 | SodA- pGL01-F | ATAGGATCCATGAGTTATACACTGCCATCC | Gene Clone |
| 2 | SodA - pGL01-R | ATACTCGAGTTATTTTTTAGCGGCGAAACGCGC | Gene Clone |
| 3 | YdiU-18C-F | ATATCTAGAATGACCCTGTCTTTTACTGCC | Gene Clone |
| 4 | YdiU-18C-R | ATAGGTACCACTTGAACAACTGACCTCCAG | Gene Clone |
| 5 | SodA -T25-F | ATATCTAGAATGAGTTATACACTGCCATCC | Gene Clone |
| 6 | SodA -T25-R | ATAGGTACCTTTTTTAGCGGCGAAACGCGC | Gene Clone |
| 7 | YdiU-F | aactggcgcagcagttag | qPCR |
| 8 | YdiU-R | gatgcccgctatatacctg | qPCR |
| 9 | SodA-F | ATCGAGCGTGACTTCGGTTC | qPCR |
| 10 | SodA-R | GCCAGTTTGTCGCCTTTCAG | qPCR |
