# Supplementary material for: Oxidative stress-triggered UMPylation of SodA by YdiU modulates oxidative stress resistance in Salmonella
Source: Vet Res. 2026 Jul 11;57:131. doi: 10.1186/s13567-026-01818-7 (PMC13355359; doi:10.1186/s13567-026-01818-7)
Supplement: Supplementary file 3 — Additional file 3 Plasmids used in this study. [file 13567_2026_1818_MOESM3_ESM.docx]

**Plasmids used in this study**

| No | Plasmids | Relevant characteristic(s) | Source |
| --- | --- | --- | --- |
| 1 | YdiU^475^/pGL01 | YdiU 1-475aa cloned into pGL01 | (Yang et al., 2020) |
| 2 | pGL01 | Expression Vector Amp^+^ | (Li et al., 2012) |
| 3 | SodA /pGL01 | SodA full-length cloned into pGL01 | This study |
| 4 | pET29b | Expression Vector Kan^+^ | Novagen |
| 5 | YdiU^475^/pET29b(3T) | YdiU 1-475aa cloned into pET29b | (Yang et al., 2020) |
| 6 | SodA /pKNT25 | FlhDC full-length cloned into pKNT25 | This study |
| 7 | YdiU /pUT18C | YdiU full-length cloned into pUT18C | (Ma et al., 2022) |
