# Supplementary figures and images for: Oxidative stress-triggered UMPylation of SodA by YdiU modulates oxidative stress resistance in Salmonella
Source: Vet Res. 2026 Jul 11;57:131. doi: 10.1186/s13567-026-01818-7 (PMC13355359; doi:10.1186/s13567-026-01818-7)

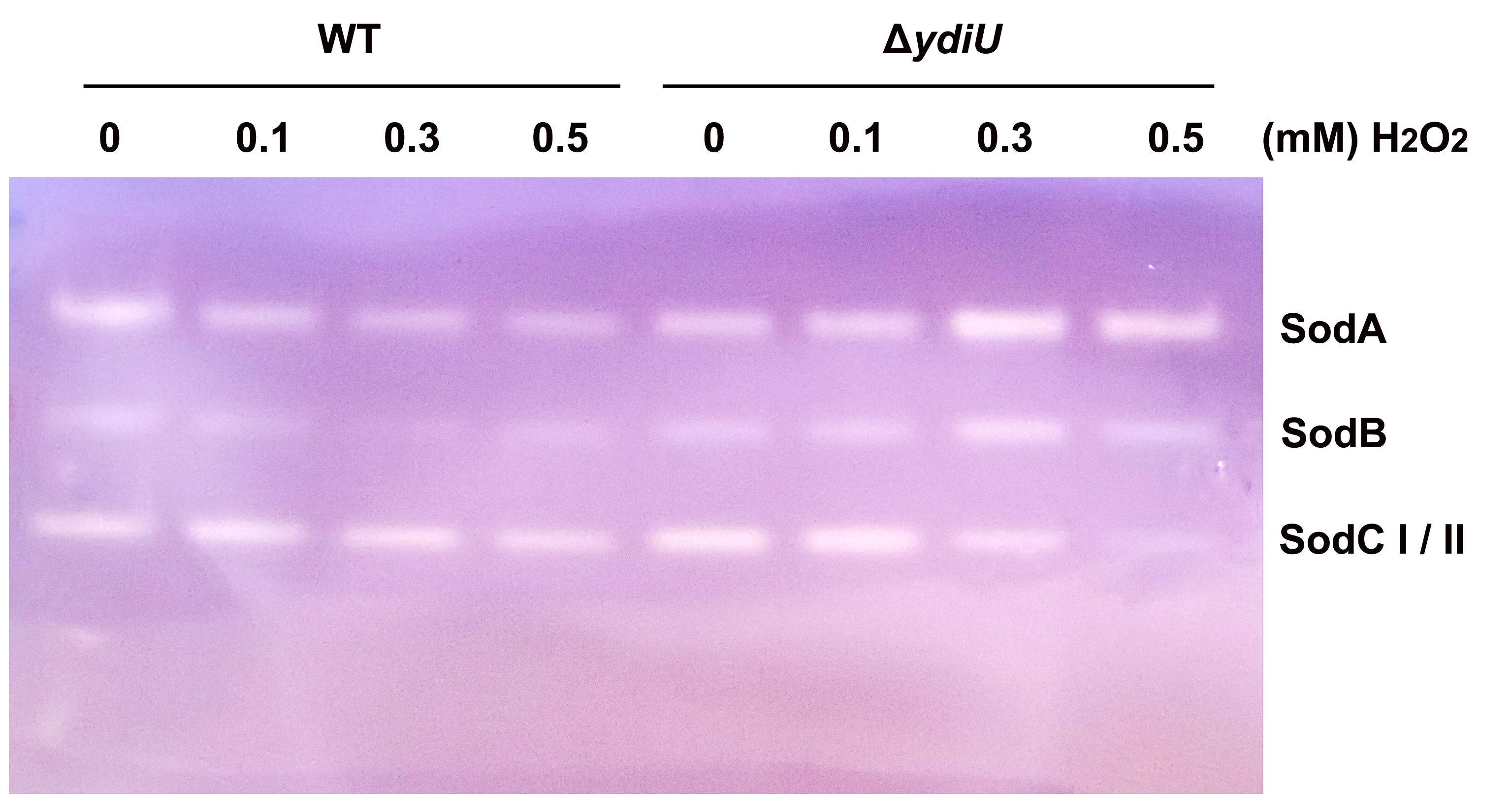

Supplement: Supplementary file 4 — Additional file 4 In-gel SOD isoform activity profiling in WT and ΔydiU S. Typhimurium under H₂O₂-induced oxidative stress. SOD enzymatic activities were resolved by native polyacrylamide gel electrophoresis (Native-PAGE) followed by nitroblue tetrazolium (NBT)-riboflavin-based in-gel activity staining. Strains examined included the WT S. Typhimurium and an isogenic ΔydiU deletion mutant. Bacterial cells were grown to an optical density of 0.5 at 600 nm (OD₆₀₀) in medium containing 0, 0.1, 0.3, or 0.5 mM H2O2, and subsequently harvested and lysed. Three distinct, reproducible SOD activity bands were observed, ordered from highest to lowest electrophoretic mobility: SodA (Mn-SOD), SodB (Fe-SOD), and the co-migrating Cu/Zn-SOD isoforms SodCI and SodCII. [file 13567_2026_1818_MOESM4_ESM.tif]

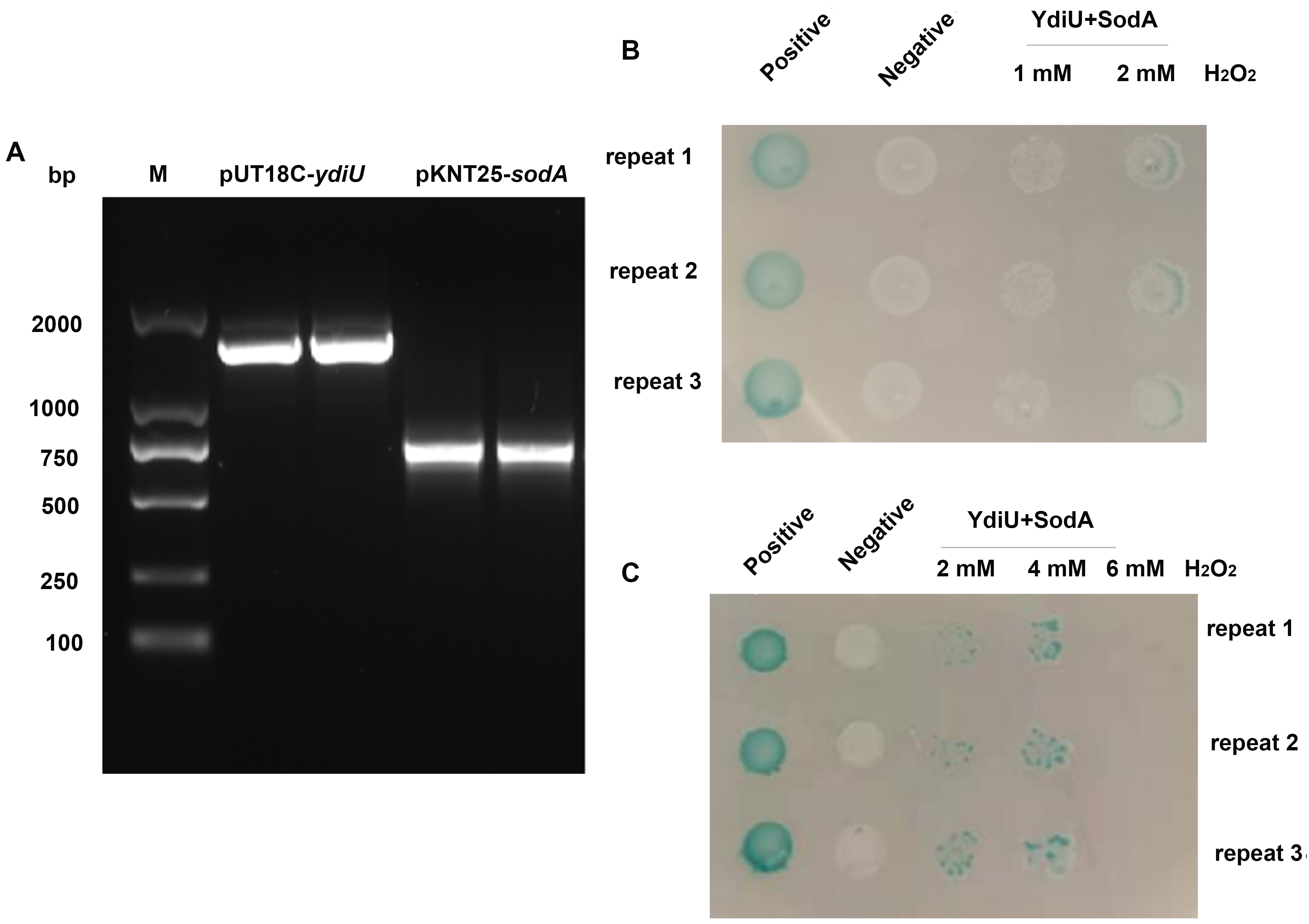

Supplement: Supplementary file 5 — Additional file 5 The interaction between YdiU and SodA. (A) Identification of positive recombinant strains pUT18C-ydiU, pKNT25-sodA by DNA electrophoresis. (B) Direct interaction between YdiU and SodA was detected by a bacterial two-hybrid method. Positive control: recombinant strain BTH101 containing T18-zip and T25-zip vectors. Negative control: recombinant strain BTH101 containing the empty T18 and T25 vectors. 1 and 2 represented the interaction betweenYdiU and SodA under normal circumstances and 2 mM H2O2 oxidative stress, respectively. (C) Direct interaction between YdiU and SodA was detected by a bacterial two-hybrid method under 2, 4 and 6 mM H2O2 stress, respectively. [file 13567_2026_1818_MOESM5_ESM.tif]

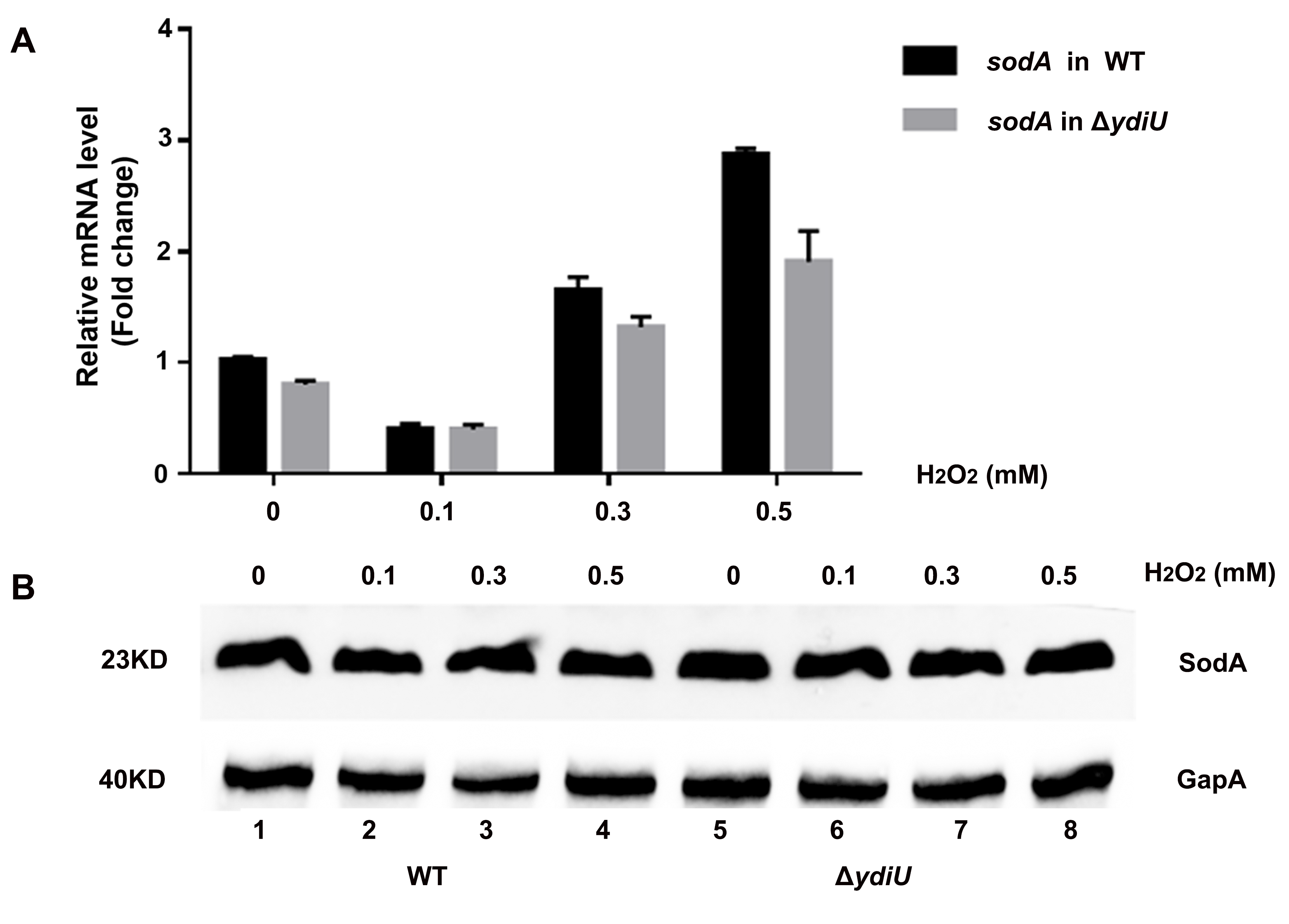

Supplement: Supplementary file 6 — Additional file 6 The expression level of SodA with different concentrations of H2O2. (A and B) The transcription and protein levels of SodA in Salmonella cultivated with different concentrations of H2O2 were detected with qRT-PCR and western blotting, respectively. GapA (also known as glyceraldehyde-3-phosphate dehydrogenase [GAPDH]) was used as a loading control. Lane 1-4: WT, Lane 5-8: ΔydiU. [file 13567_2026_1818_MOESM6_ESM.tif]

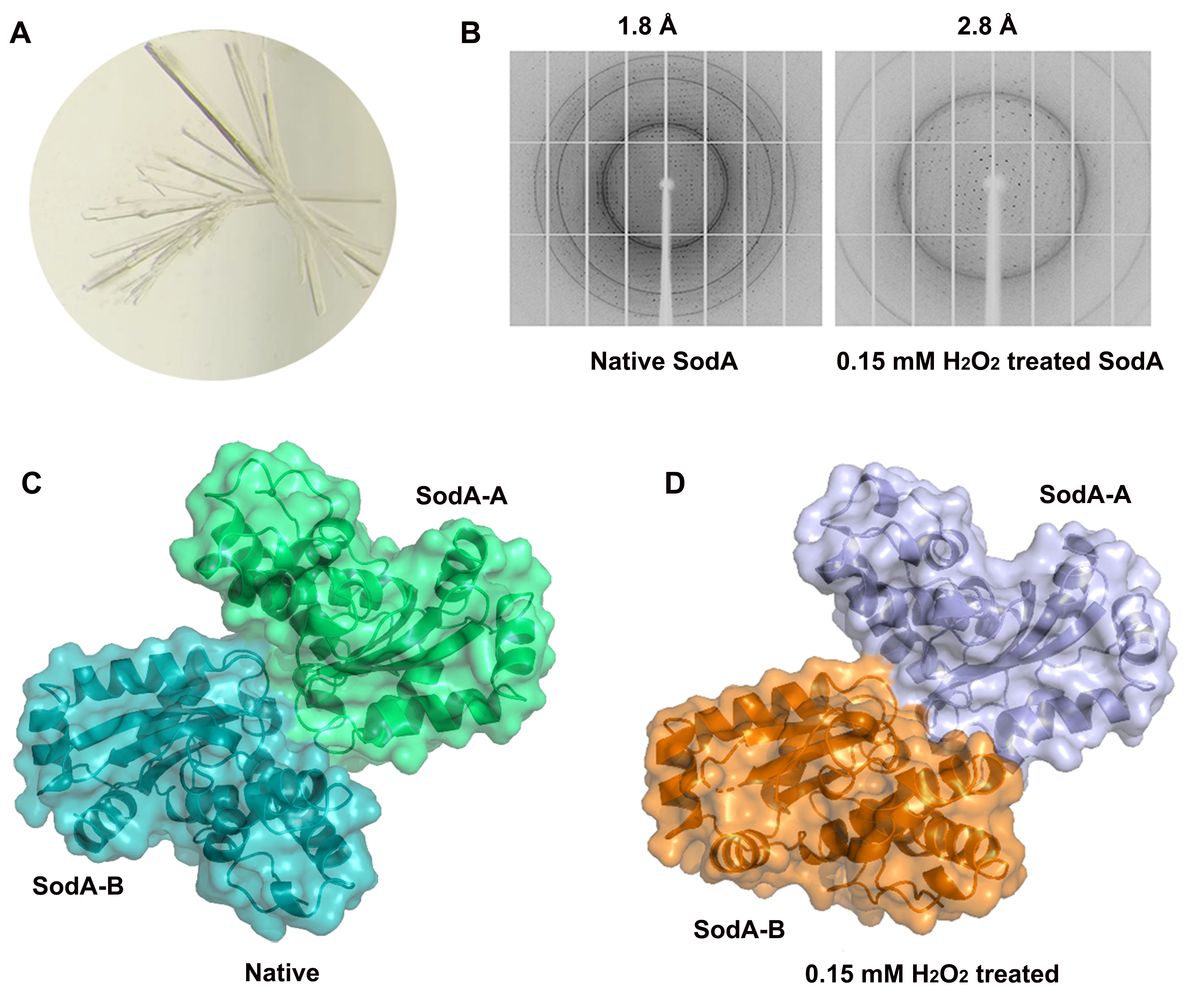

Supplement: Supplementary file 7 — Additional file 7 Crystal structure analysis of native SodA and 0.15 mM H2O2 treated SodA. (A) The crystal shapes of the native SodA and 0.15 mM H2O2 treated SodA were similar. (B) Protein diffraction data were received in Shanghai Light Source, and the resolution of native SodA and 0.15 mM H2O2 treated SodA was 1.8 Å, 2.8 Å, respectively. (C) Crystal structures of native SodA. (D) Crystal structures of 0.15 mM H2O2 treated SodA. [file 13567_2026_1818_MOESM7_ESM.tif]

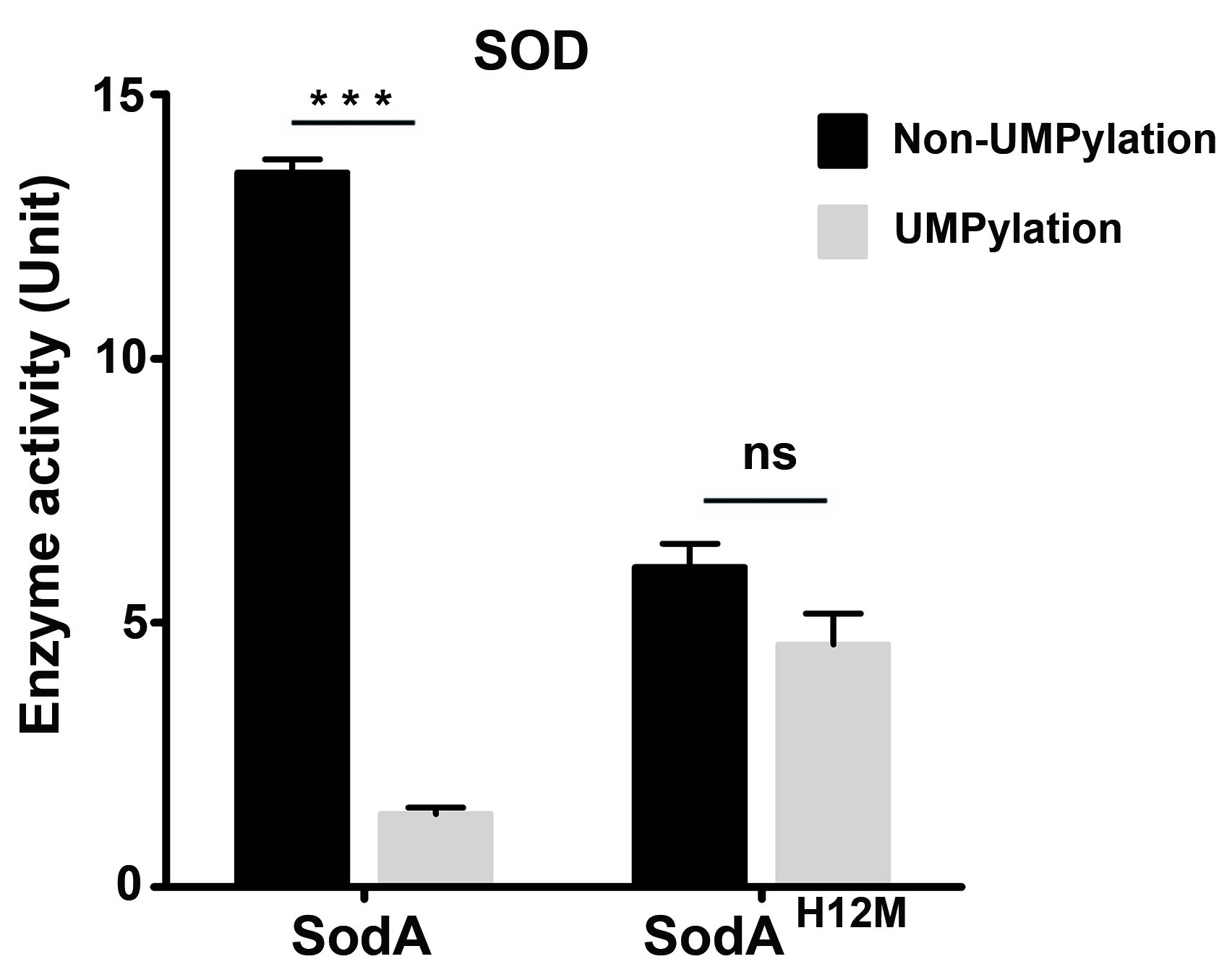

Supplement: Supplementary file 8 — Supplementary Material 8. Additional file 8 Impact of UMPylation on the Enzymatic Activity of native SodA and the H12M variant. Enzymatic activities of native SodA and its H12M point mutant were quantified under both unmodified and UMPylated conditions. Data are presented as mean ± standard deviation (n = 3 independent experiments). Statistical significance was determined using unpaired two-tailed Student’s t-test. ***p < 0.001; ns, no significant difference (p ≥ 0.05). [file 13567_2026_1818_MOESM8_ESM.tif]
